# Wide Screening of Phage-Displayed Libraries Identifies Immune Targets in Planta

**DOI:** 10.1371/journal.pone.0054654

**Published:** 2013-01-25

**Authors:** Cristina Rioja, Saskia C. Van Wees, Keith A. Charlton, Corné M. J. Pieterse, Oscar Lorenzo, Susana García-Sánchez

**Affiliations:** 1 Dpto. de Fisiología Vegetal, Centro Hispano-Luso de Investigaciones Agrarias (CIALE), Universidad de Salamanca, Salamanca, Spain; 2 Instituto Vasco de Investigación y Desarrollo Agrario NEIKER-Tecnalia, Vitoria-Gasteiz, Spain; 3 Department of Biology, Plant-Microbe Interactions, Utrecht University, Utrecht, The Netherlands; 4 Scotia Biologics Ltd., Aberdeen, United Kingdom; 5 Functional Genomics Unit, CIC-Biogune, Derio, Spain; 6 Department of Physiology, University of Basque Country-IKERBASQUE, Leioa, Spain; AC Camargo Cancer Hospital, Brazil

## Abstract

Microbe-Associated Molecular Patterns and virulence effectors are recognized by plants as a first step to mount a defence response against potential pathogens. This recognition involves a large family of extracellular membrane receptors and other immune proteins located in different sub-cellular compartments. We have used phage-display technology to express and select for Arabidopsis proteins able to bind bacterial pathogens. To rapidly identify microbe-bound phage, we developed a monitoring method based on microarrays. This combined strategy allowed for a genome-wide screening of plant proteins involved in pathogen perception. Two phage libraries for high-throughput selection were constructed from cDNA of plants infected with *Pseudomonas aeruginosa* PA14, or from combined samples of the virulent isolate DC3000 of *Pseudomonas syringae* pv. *tomato* and its avirulent variant *avrRpt2*. These three pathosystems represent different degrees in the specificity of plant-microbe interactions. Libraries cover up to 2×10^7^ different plant transcripts that can be displayed as functional proteins on the surface of T7 bacteriophage. A number of these were selected in a bio-panning assay for binding to *Pseudomonas* cells. Among the selected clones we isolated the ethylene response factor ATERF-1, which was able to bind the three bacterial strains in competition assays. ATERF-1 was rapidly exported from the nucleus upon infiltration of either alive or heat-killed *Pseudomonas*. Moreover, *aterf-1* mutants exhibited enhanced susceptibility to infection. These findings suggest that ATERF-1 contains a microbe-recognition domain with a role in plant defence. To identify other putative pathogen-binding proteins on a genome-wide scale, the copy number of selected-*vs.*-total clones was compared by hybridizing phage cDNAs with Arabidopsis microarrays. Microarray analysis revealed a set of 472 candidates with significant fold change. Within this set defence-related genes, including well-known targets of bacterial effectors, are over-represented. Other genes non-previously related to defence can be associated through this study with general or strain-specific recognition of *Pseudomonas*.

## Introduction

The interactions between plants and micro-organisms in nature are complex and diverse. Microbes can be potential pathogens or beneficial partners, and plants have developed sophisticated mechanism to detect and neutralize them or to make use of their metabolism. Conversely microbes have evolved mechanisms to evade plant immune systems and to use plants as nutritional reservoirs. This co-evolution of plants with micro-organisms has lead to the occurrence of different families of molecules involved in microbial recognition.

Microbe-associated molecular patterns (MAMPs) are structural components of the microbes that can be recognized by the plant and induce pattern-triggered immunity (PTI), the first immune barrier [1]–[3]. MAMPs are conserved within specific microbial families and include diverse molecules such as flagellin, lipopolysaccharide (LPS), fungal chitin or the bacterial EF-Tu elongation factor [4]–[6]. Their recognition is also essential for the establishment of beneficial interactions, a process that is coupled to the suppression of PTI [7], [8]. Plants perceive the different types of MAMPs through specific pattern-recognition receptors (PRRs), the best known of which include LRR (Leucin-Rich Repeat) receptor-like proteins (RLPs) and receptor-like kinases (RLKs) [9]. These families of receptors are also found in mammals. In contrast to mammals, plant genomes contain hundreds of genes encoding for RLK and RLP proteins [10], [11]. In addition, there are many “orphan” MAMPs that are known to elicit immune response in plants but for which the specific receptors have not yet been discovered. Successful pathogens have also evolved virulence effectors to interfere with PTI and render the plant susceptible to infection. *Pseudomonas syringae* for example, produces more than 30 different effectors that are secreted upon contact with host plants and target PTI components [12], [13]. As a counterpart, plants have developed corresponding resistance (R) proteins to recognize these effectors and their modified targets, which results in effector-triggered immunity (ETI) [2]. ETI also involves specific families of plant proteins, notably nucleotide-binding-LRR (NB-LRR) proteins [14], which are believed to integrate effector perception and activation of immune-inducible genes through interactions between their modular domains [15].

Although PTI and ETI responses trigger different defence mechanisms in plants, the distinction between both types of immunity is not always clear [16], [17]. The diversity of MAMPs or virulence effectors that microorganism can display and the multiplicity of the LRR-type receptors that are encoded in plant genomes suggest that a large number of plant proteins could participate in the recognition of bacterial molecules. In this regard, high-throughput protein-interaction screenings are suitable to determine which plant proteins can function as immune receptors for microbial ligands [17], [18]. As an example, by using a yeast two hybrid-based pipeline an interaction network with different pathogen effectors has been created that includes more than 8,000 Arabidopsis proteins [18].

Phage display has been used for more than twenty five years as a powerful tool to discover protein-ligand interactions [19]–[21]. With this technique, peptides or proteins are functionally displayed on a viral surface as fusions with viral coat proteins, and ligands of interest are used to select for interacting partners. Since the displayed protein and its encoding gene are physically linked in the same viral particle, the identification of selected proteins only requires nucleic acid sequencing. Another key feature of this technology is that allows for the display of large numbers (up to ∼10^11^) of peptide variants. Individual phage clones are selected from billions of different phage particles on the basis of the binding affinity of their displayed protein for the ligand of choice; selected clones are then amplified and the process iterated to enrich the initial phage population in affinity-binding clones. This so-called bio-panning selection can be manipulated to result in a fine tuning of protein-ligand interaction in the presence of competitive partners.

The possibility of selecting strong protein-ligand interactions between competing partners made phage display a widely-used technology to discover high-affinity antibodies [22]. In addition, the versatility of phage libraries and bio-panning techniques makes the technology suitable for the isolation of a variety of naturally occurring proteins which interact with their physiological ligands. cDNA libraries displayed in phage particles have been used to identify natural protein complexes in a similar way to two-hybrid screening or to discover *in vivo* interactions by injection into living animals and recovery of targeted organs [23].

In this paper we constructed two phage-display libraries from the cDNA of microbe-challenged Arabidopsis. Recombinant phage displaying plant proteins capable of interacting with different species of *Pseudomonas* were selected by bio-panning using microbial cells as selection ligands. Selected phage were identified by two approaches *i)* sequencing of the dominant clones isolated after bio-panning and *ii)* hybridization of total *vs.* selected cDNAs to Arabidopsis microarrays. The latter was used to compare microbe-binding properties of selected clones on a genome-wide scale. We identified plant proteins involved in defence response and confirmed *in vitro* its capacity to bind microbial cells. The use of different strains of *Pseudomonas* allowed us to discern between common bacterial receptors and specific targets of virulent or avirulent strains.

## Results

### 1 Construction of Arabidopsis cDNA libraries for phage-display

We used two species of *Pseudomonas* to elicit immune response in Arabidopsis. This genus includes very ubiquitous bacteria able to parasite a wide range of hosts. *P. aeruginosa* is typically an opportunistic pathogen of humans which also infects other vertebrates, insects and plants [24], whereas *P. syringae* is a natural pathogen of plants with different host-specific pathovars. Both species share common MAMPs and effectors [24], [25] but differ in their adaptation to specific host biology and defence mechanisms [12], [26]. The library T7LAtPa was constructed with cDNA obtained from plants infected with the PA14 strain of *P. aeruginosa* (*Pa*) [27], [28]. In this infection model, plants were grown in liquid medium and bacteria were inoculated as described by these authors; mRNA was purified at different times post-infection and pooled for cDNA preparation. For the T7LAtPs library plants were grown in soil and infected with *P. syringae* pv. *tomato* by infiltration of bacteria into plant leaves; plants were infiltrated either with the virulent strain DC3000 (*Pst*) or its avirulent variant *Pst*(*avrRpt2*) [29] and cDNAs were pooled before the cloning step. In both cases an early defence response was observed in the plant as chlorotic lesions appearing on the infected tissues. The infection with *Pa* or *Pst* strains progressed until plant death, whereas *Pst(avrRpt2)* only caused a hypersensitive reaction without further damage for the host.

Plant cDNAs were cloned into the T7Select10-3 vector. This system uses the T7-10B capsid protein of lytic phage to display foreign polypeptides of up to 1200 amino acids. cDNAs that are cloned in frame with the 3′ end of the T7-10B-encoding gene (about 1/3 of the fusions) can be displayed as recombinant proteins on the viral surface. The recombinant phage genomes generated after cDNA ligation into the vector were *in vitro* packaged to generate a primary library, which was next transfected into the *E .coli* host to allow for replication and translation of recombinant capsids. Transfected cultures were plated with molten agarose to determine the number of plaque forming units (pfu) in the primary library and then amplified to 10^10^–10^11^ pfu/ml. These amplified suspensions constituted our stock libraries for further bio-panning experiments. To estimate the complexity of the libraries we analyzed the inserts contained in a representative fraction of amplified viral clones (Table 1). According to this estimation, the T7LATPa library contained at least 7.7×10^4^ different cDNA inserts, whereas the T7LATPs library, where the size and the proportion of clones with insert were higher, contained 2.3×10^7^. Considering that 1/3 of the cloned cDNAs are expected to be in-frame fusions, both libraries should contain at least one in-frame cDNA fragment representative for each protein encoded in the *A. thaliana* genome (about 2.5×10^4^ protein-encoding genes). Further hybridizations of the labelled cDNA inserts with high-coverage microarrays for Arabidopsis confirmed a wide representation of genes in the T7LAtPs library (Figure S1). Hybridization of this library produced detectable signals for 24,836 out of the 29,110 gene elements spotted in the microarray, whereas 13,720 elements were detected in the T7LAtPa library.

**Table 1:** Characteristics of the phage-display libraries constructed for this study.

	Library	
Parameter	T7LAtPa	T7LAtPs
Microorganism infected in Arabidopsis	*P. aeruginosa*	*P. syringae*
Total pfu in primary library (**a**)	7×10^5^	6×10^7^
Clones with insert (**b**)	20%	70%
Insert sizes (kb)	0.2–1.5	0.2–2.2
Non-redundant inserts (**c**)	55%	55%
Most redundant insert	5.6%	16%
Total number of non-redundant inserts (**d**)	7.7×10^4^	2.3×10^7^

**d** = **a**×**b**/100×**c**/100.

### 2 Selection of *Pseudomonas*-bound clones by bio-panning

**Figure 1:**
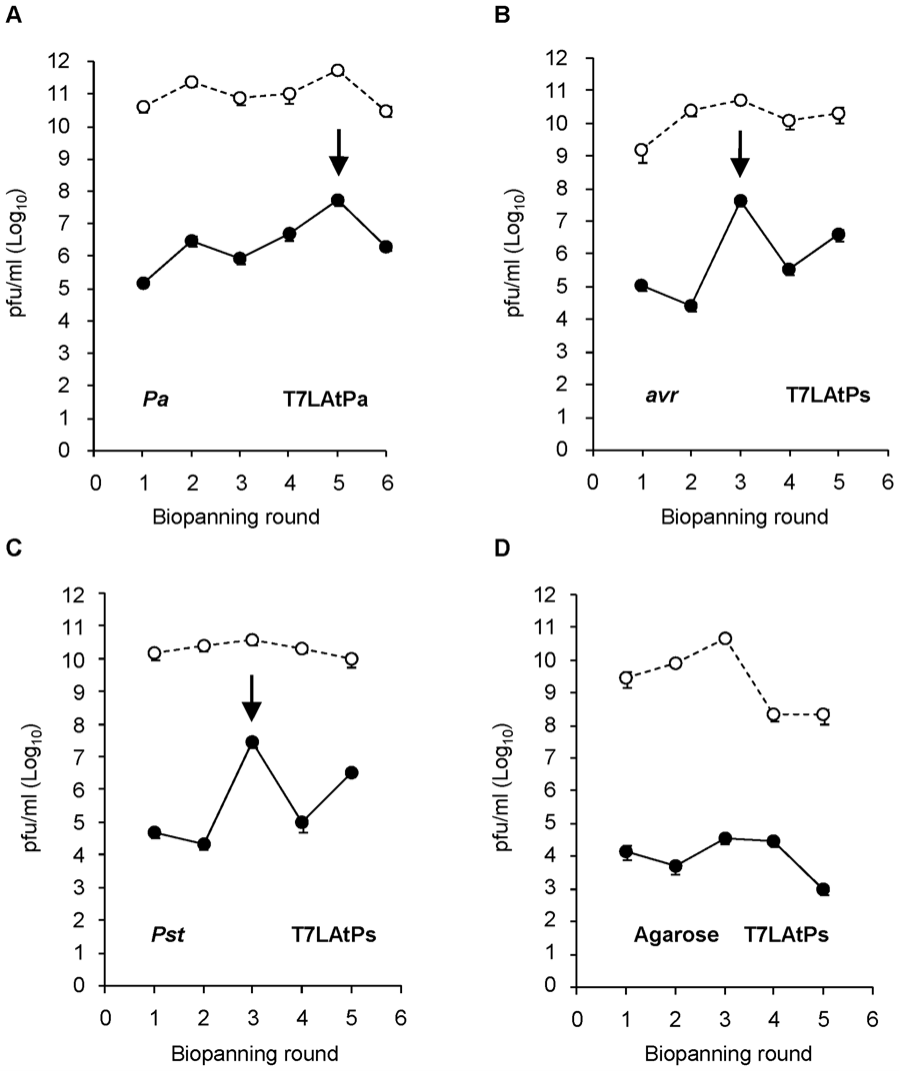
Titres of eluted phage increase after successive rounds of bio-panning with *Pseudomonas sp*. Bacteria were pre-incubated with 10-to-15-days old plantlets in liquid MS medium and collected from the roots before bio-panning. Phage (6.3×10^9^ pfu from T7LAtPa or T7LAtPs libraries) were panned against 1 OD of (**A**) *Pa* (**B**) *Pst(avrRpt2)* (*avr*) (**C**) *Pst* or (**D**) agarose (Agar). After washes, bound phage were eluted and amplified for the next round of bio-panning. The titres of eluted (black dots, continuous line) or amplified (white dots, dashed line) phage were calculated by pfu-counting. The arrows indicate the peak of maximal titre for each pan series.

Both libraries were panned to select for phage clones displaying candidate targets for MAMPs or virulence effectors. Since some effectors might be expressed by the pathogen only upon contact with the host, bacterial cells were handled as in the root infection model described for PA14 strain [28]. Bacteria were incubated together with *A. thaliana* plantlets in liquid MS medium and recovered alive from plant surfaces before using them as the ligands for selection. Bio-panning was performed by incubating the amplified phage libraries (6.3×10^9^ pfu) with these infective cells of *Pa* (T7LAtPa library), *Pst* or *Pst*(*avrRpt2*) strains (T7LAtPs library in both cases). *Pseudomonas*-bound phage were recovered by elution from bacterial pellets and re-amplified to 6.3×10^9^ pfu for successive rounds of selection. Eluates were titred after each round to assess for the enrichment in specific-binding clones (Figure 1). Bio-panning of the T7LAtPa library with *Pa* cells produced eluates with an initial titre of 1.5×10^5^ pfu/ml (in the first round), which increased up to a maximum of 5×10^7^ pfu/ml in the 5^th^ round. Bio-panning of the T7LAtPs library with *Pst* or *Pst*(*avrRpt2*) cells produced titres similar to those of *Pa* in the first round, but reached a maximum more rapidly (after 3 rounds 4.3×10^7^ pfu/ml for *Pst*(*avrRpt2*) and 2.8×10^7^ pfu/ml for *Pst*). However bio-panning with agarose beads as a control for non-specific binding failed to produce high eluate titres, with the number of eluted phage falling down to 10^3^ pfu/ml in the 5^th^ round. These data suggested that enrichment in specific-binding clones already happened in rounds 5^th^ (*Pa*) and 3^rd^ (*Pst*(*avrRpt2*) and *Pst*), and additional rounds of amplification and selection were not required.

### 3 Sequence analysis of dominant clones selected by bio-panning

The pool of cDNA inserts contained in the eluates from maximum-titre rounds (the 5^th^ round for *Pa* or 3^rd^ round for *Pst* and *Pst*(*avrRpt2*) strains) was PCR-amplified using oligonucleotides in the flanking regions of the T7-10B gene. The cDNA amplicon from the panned eluates was enriched in specific bands when compared to the “smear” from non-panned libraries (not shown). Thus, the increase of the eluate titres after bio-panning correlated with enrichment in certain types of cDNA inserts, which strengthened the hypothesis of specific selection occurring between these rounds.

**Figure 2:**
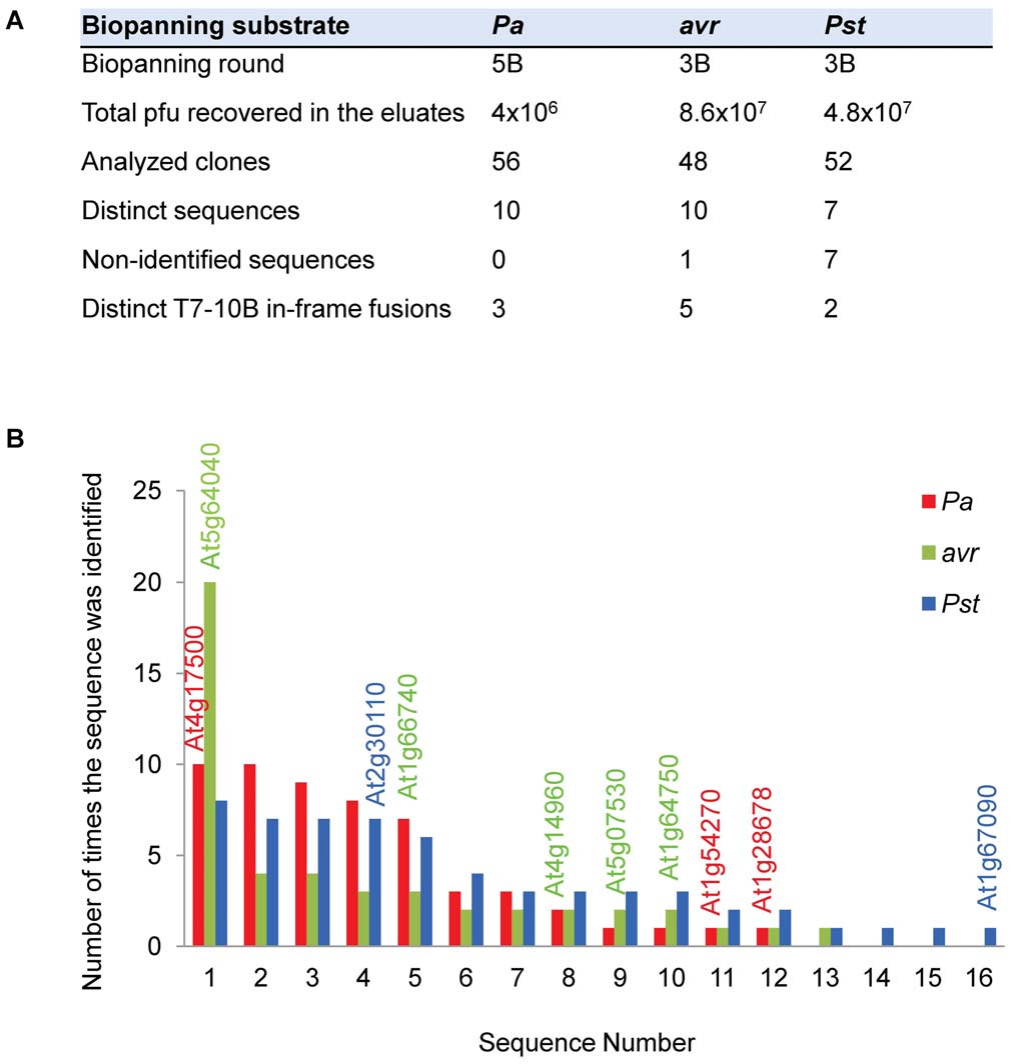
Sequence analysis of 156 phage clones eluted after bio-panning with *Pa*, *Pst*(*avrRpt2*) (*avr*) or *Pst* strains. (**A**) Number of phage clones analyzed from each selection. (**B**) Bar graph shows the redundancy of the sequences that were identified. The 10 sequences containing T7-10B in-frame fusions are labelled with their TAIR accession on the top of the corresponding bars.

The eluates from enriched rounds contained between 4×10^6^ and 4.8×10^7^ pfu (Figure 2A). To identify the dominant clones selected, the eluates were diluted and plated, and individual pfu were randomly picked to sequence their cDNA inserts. A total of 156 clones from the 3 bio-pannings were analyzed. As expected, most inserts were present as redundant copies and only 27 different sequences were found (Figure 2A, B). The study of their fusion sites resulted in the identification of 10 different polypeptides in frame with the T7-10B minor coat protein. Their description and the fraction of the full-length protein that is fused to T7-10B are shown in Table 2. The most abundant clone rescued from the *Pa* bio-panning encoded for the defence-related protein ATERF-1 (At4g17500), a member of the AP2/ERF-family of transcription factors which is highly induced upon infection with different pathogens [30]–[32]; the ATERF-1 fusion to T7-10B (amino acids 176 to 268) comprises the DNA-binding and defence-related domains. The PSAN subunit of photosystem I (At5g64040) and the anti-silencing protein AtSP7 (At1g66740) were the dominant clones identified from *Pst*(*avrRpt2*) bio-panning and both contained significant fragments of the full-length proteins (82% and 83% respectively). The major in-frame clone rescued from *Pst* bio-panning encoded for the ubiquitin-activating enzyme ATUBA1 (At2g30110). This protein has a role in defence, since a 15-bp deletion in its C-terminus (*mos5* mutant) is able to revert the constitutive defence response phenotype of *snc1* mutant [33]. Interestingly, the fusion with the T7-10B protein in the rescued clone covers the fragment of ATUBA1 that is deleted in *mos5* (amino acids 1040 to 1080).

**Table 2:** Sequence homology of the 10 in-frame clones selected after bio-panning.

Bio-panning substrate	Gene ID	Description	Aminoacids fussed to T7-10b	Percentage of the full-length	Frequency of isolation
***Pa***	At4g17500	ATERF-1; Ethylene Responsive Element Binding Factor 1	176–268	35%	10/56
	At1g54270	EIF4A-2; Eukaryotic Translation Initiation Factor 4A-2	175–412	58%	1/56
	At1g28670	ARAB-1; Carboxylesterase/Hydrolase, acting on ester bonds	316–384	18%	1/56
***avr***	At5g64040	PSAN; Calmodulin Binding	30–171	82%	20/48
	At1g66740	AtSP7; Anti Silencing Protein 7	34–196	83%	3/48
	At1g64750	ATDSS1(I); Arabidopsis thaliana Deletion of SUV3 Supressor 1 (I)	40–74	46%	2/48
	At5g07530	GRP17; Glycine Rich Protein 17	161–512	69%	2/48
	At4g14960	TUA6; Structural Constituent of Cytoskeleton	391–427	8%	2/48
***Pst***	At2g30110	ATUBA1; Ubiquitin Activating Enzyme/ubiquitin-protein ligase	1040–1080	4%	7/52
	At1g67090	RBCS1A; Ribulose Bisphosphate Carboxylase Small Chain 1A	101–136	26%	1/52

### 4 T7-ATERF-1 clone binds competitively to *Pseudomonas* cells

**Figure 3:**
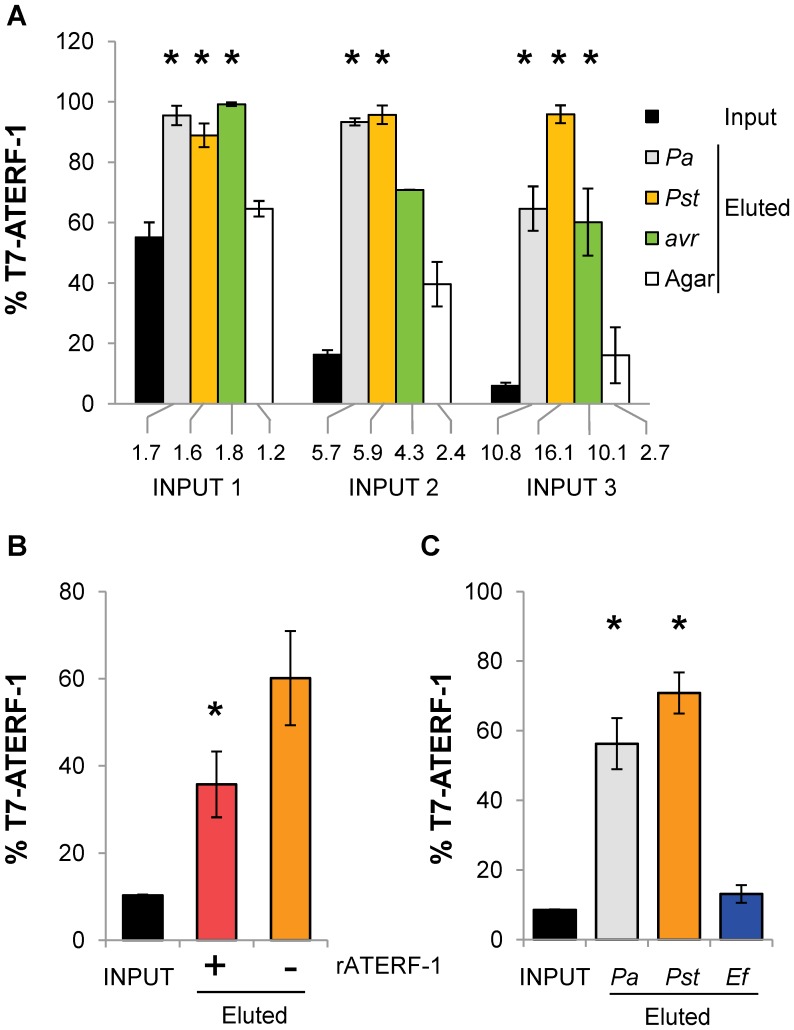
Competitive bio-panning assay shows specific binding of T7-ATERF-1 clone to the three strains of *Pseudomonas*. (**A**) Competition between T7-ATERF-1 phage (which expresses ATERF-1 polypeptide) and T7-C1 (competing phage randomly selected). Mixtures of phage containing 1∶1 (input 1), 1∶6 (input 2) and 1∶17 (input 3) proportions of T7-ATERF-1:T7-C1 clones were prepared to a final concentration of 6.3×10^9^ pfu/ml. Phage mixtures were panned against 1 OD of *Pa*, *Pst*, *Pst*(*avrRpt2*) (*avr*) or agarose (Agar) under the same conditions used for T7LAtPa and T7LAtPs libraries. The bar series show the percentage of T7-ATERF-1 clone in the input and in the eluates that were recovered after one round of selection. The numbers below the bars represent the fold enrichment for T7-ATERF-1, calculated as the ratio between eluate and input percentages. Asterisks indicate significant differences *(t*-test, p<0.05) respect to the agarose (non-specific binding) control. (**B**) Addition of rATERF-1 protein inhibits binding of T7-ATERF-1 phage to *Pst* cells. Bacteria were incubated with 10 µg of rATERF-1 (+) or rLacZ (−) during panning with a 1∶10 mixture (input) of T7-ATERF-1:T7-C1 phage. Recovery of T7-ATERF-1 in the eluates was significantly decreased (p<0.05) when rATERF-1 was added to the cells. (**C**) Competition between T7-ATERF-1 and T7-C1 clone for binding to *Pa*, *Pst* or *Enterococcus faecalis* (*Ef*). The percentage of T7-ATERF-1 phage recovered from bio-panning with the Gram(+) bacterium was not significantly different from the input (p = 0.1835), whereas eluates recovered from *Pseudomonas sp.* bio-pannings were enriched in T7-ATERF-1 clone (*Pa* p = 0.0086 and *Pst* p = 0.0021).

To confirm the *Pseudomonas*-binding capacity of the proteins selected by our bio-panning method, we performed competitive bio-panning assays for two dominant clones from Table 2 (T7-ATERF-1 and T7-ATUBA1). Phage displaying the ATERF-1 polypeptide (T7-ATERF-1 clone) or a competing, non-related peptide (T7-C1 clone), were bio-panned against bacterial cells. The T7-ATERF-1:T7-C1 phage clones were mixed in three different input proportions (1∶1, 1∶6 and 1∶17) and bio-panned against the three strains of *Pseudomonas* or against agarose as a control for non-specific binding (Figure 3A). In the three cases after a single round of bio-panning the mixture recovered from the eluates contained T7-ATERF-1 as the major clone, representing up to 100% of the rescued phage. In contrast, the proportion of this clone recovered after bio-panning against agarose remained similar to the input mixture. Using the minimal input (1∶17) as the baseline, the ratio between input and rescued phage was used to assess for the maximal enrichment in T7-ATERF-1 that could be detected after one round of selection. The three strains resulted in significant and more-than-10-fold enrichment. To exclude the possibility that a mutation in the phage particle rather than the phage-displayed polypeptide itself was responsible for increased binding, recombinant (r) ATERF-1 protein was added to the cells during bio-panning. The addition of rATERF1 reduced recovery of T7-ATERF-1 clone to less than 40% (Figure 3B). This suggests that rATERF-1 can compete with the T7-ATERF-1 polypeptide for the binding, resulting in less T7-ATERF-1 phage bound to the cells during bio-panning. These results support the assertion that ATERF-1 protein binds selectively to *Pseudomonas* cells. This binding is likely through a bacterial component common to the three strains, since panning against all of them resulted in a significant increase of the T7-ATERF-1 clone in rescued phage. To further demonstrate the specificity of T7-ATERF-1 binding, a Gram (+) bacteria was used as the substrate for competitive bio-panning under identical conditions than *Pseudomonas* strains (Figure 3C), and was found unable to produce significant enrichment. Since LPS is a MAMP common to all Gram (−) bacteria and it has been demonstrated that induces expression of a high number of defence-related genes in *A. thaliana* [5], bio-panning was also performed against immobilised bacterial LPS (Figure S2A). Although bio-panning against LPS resulted in a considerable enrichment in recovered T7-ATERF1 phage, *t*-test comparison produced high p-values and the result was considered non significant.

A similar competition assay was performed for the clone T7-ATUBA1 (Figure S2B), but the maximal enrichment that could be measured in this case was 1.7-fold. Thus, we focused our next studies in the *in vivo* interactions of ATERF-1 protein during bacterial infection.

### 5 ATERF-1 is exported from the nucleus after challenge with *Pseudomonas*

**Figure 4:**
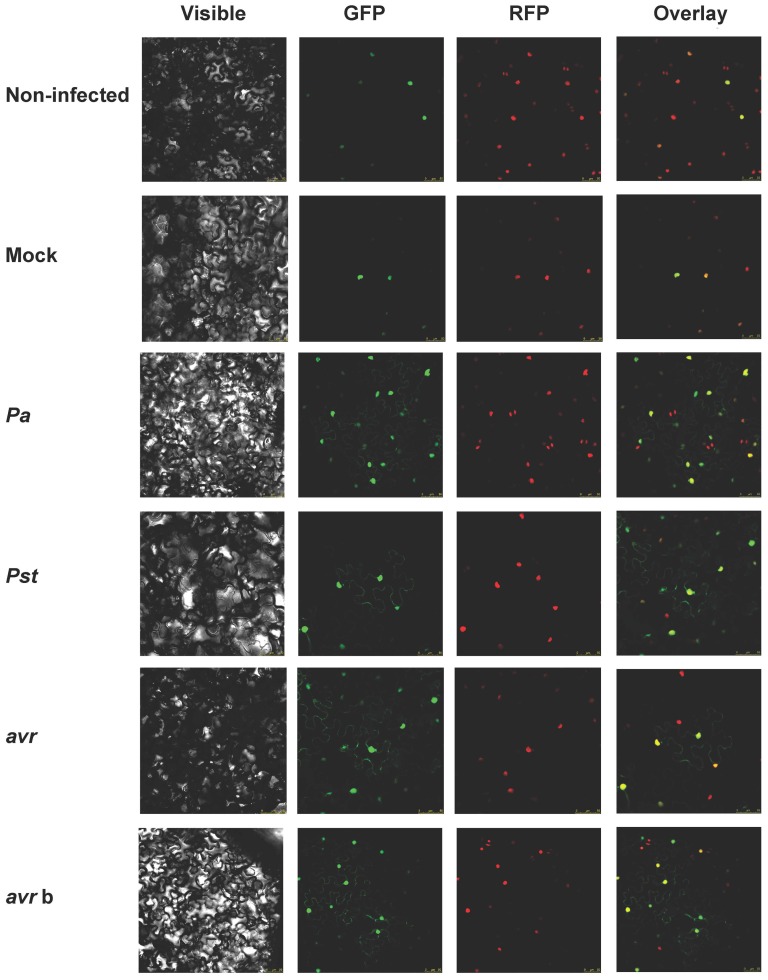
*GFP-ATERF-1* localisation after inoculation with *Pseudomonas* strains. Confocal microscopy of *N. benthamiana* leaves over-expressing *GFP-ATERF-1* together with a RFP-tagged nuclear marker (*RFP-H2B* transgenic line). Non-infected control and plants inoculated with an sterile solution of MgS0_4_ (Mock) or with a bacterial suspension of *Pa*, *Pst*, *Pst*(*avrRpt2*) (*avr*) or boiled *avr* (*avr* b) strains. Photographs were taken 3–5 h. after the inoculation.

**Figure 5:**
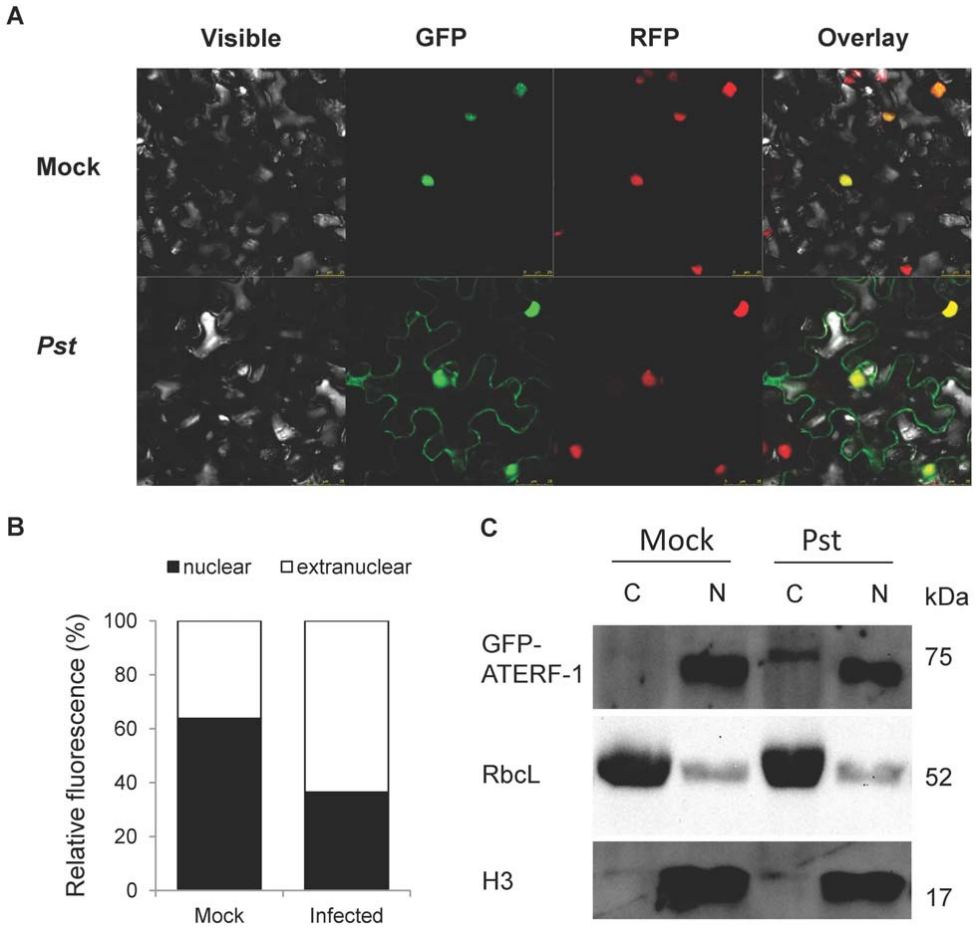
Comparison between nuclear and extra-nuclear *GFP-ATERF-1* in non-infected and *Pst* infected plants. (**A**) Higher magnification photographs (25 µm scale) show extra-nuclear location of *GFP-ATERF-1* in *Pst*-infected tobacco plants. (**B**) Quantification of nuclear and extra-nuclear GFP fluorescence from a total of 203 cells, in non-infected and infected tobacco plants. (**C**) Western-blot detection of GFP protein in cytoplasmic and nuclear fractions from *35S:GFP-ATERF-1* transgenic plants of *A. thaliana*. Plants were expossed to mock or *Pst* infection and their protein extracts were separated in nuclear (N) and cytoplasmic (C) fractions. The fractions were resolved in SDS-PAGE gels, blotted and probed against anti GFP antibodies to detect the 75 KDa, *GFP-ATERF-1* fusion protein. Membranes were re-probed with anti-H3 histone (H3, nuclear) and anti RuBisCo (RbcL, cytoplasmic) antibodies.

Since the *in vivo* localization of ATERF-1 is predicted to be nuclear, the binding to bacterial components as was suggested by our competition assay is expected to take place in the nucleus as well. Bacterial MAMPs and effectors are usually perceived by plant receptors located in the plasma-membrane or internalized through the endocytic pathway to signal pathogen presence from different subcellular localisations [34]; however, very few nuclear proteins have been involved in bacterial MAMP/effector-binding [35], [36]. To determine the subcellular localization of ATERF-1, a translational fusion to the Green Fluorescent Protein (*35S:GFP-ATERF-1*) was transformed into *Agrobacterium tumefaciens* C58C1 (pGV2260) and infiltrated into *N. benthamiana* leaves, allowing transient expression of *GFP-ATERF1*. The *RFP-H2B* reporter line, which expresses nuclear Red Fluorescent Protein, was used in this study to visualize co-localization with *GFP-ATERF-1*. As expected, the localization of ATERF-1 in non-treated plants was clearly nuclear (Figure 4 and 5). However, when leaves were infiltrated with *Pa*, *Pst* or *Pst*(*avrRpt2*) strains, *GFP-ATERF-1* appeared in extra-nuclear localisations, compressed against the plasma membrane. Mock treatment failed to induce extra-nuclear localisation of *GFP-ATERF-1*, which indicates that the nuclear export is induced by the presence of bacteria rather than the wounding damage. Moreover, infiltration with heat-killed strains also resulted in extra-nuclear localisation (only shown for *Pst*(*avrRpt2*) strain), suggesting that translocation does not require metabolically active bacteria. Cytoplasmic localisation of *GFP-ATERF-1* after infiltration of *Pst* strain was confirmed by western-blot analysis in transgenic *A. thaliana* plants (Figure 5C).

### 6 Mutant *aterf-1* shows increased susceptibility to *Pst* infection

**Figure 6:**
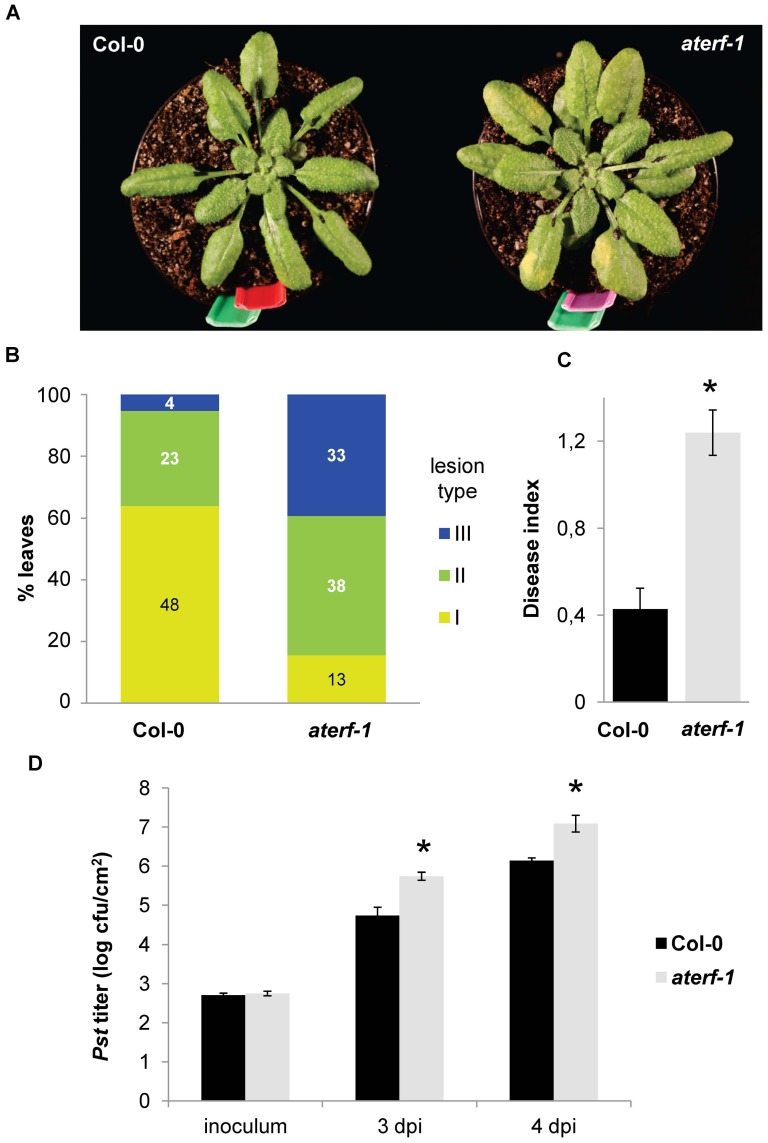
*aterf-1* mutant shows increased susceptibility to bacterial infection. (**A**) Macroscopic symptoms of disease caused by inoculation of *Pst* strain into the leaves of Col-0 and *aterf-1* mutant. (**B**) Rossette leaves of 5-week-old plants were inoculated with a *Pst* suspension in series of 8 plants per genotype. Disease symptoms (3 days post-inoculation) were rated on 75–88 leaves/genotype comparing leaves of similar developmental state. The following disease severity classes were established: I, 0–10% of leaf surface as a chlorotic lesion, II, 10–50% of leaf surface, and III, >50% of leaf surface. The percentage of leaves falling in each category is represented with the number of scored leaves inside the bars (**C**) Disease Index (DI) was calculated for each genotype according to the formula DI = (0*n*_1_+1*n*_2_+2*n*_3_)/*N*_t_ where *n*_1_–*n*_3_ is the number of leaves in the I, II or III classes and *N*_t_ is the total number of leaves collected from one plant. Asterisk indicates significant differences respect to the wild-type genotype with a p = 3.15×10^−6^ (*t*-test) (**D**) Bacterial titre in *Pst*-inoculated leaves at 3 and 4days post-inoculation (dpi). Bars represent the average cfu/cm^2^ leaf surface and error bars represent SD. Asterisks indicate significant difference between genotypes (p<0.01), which was observed in two independent bioassays.

To gain further insight into the role of ATERF-1 in plant defence, we examined the susceptibility phenotype of a loss-of-function, *aterf-1* mutant upon infection with *P. syringae*. A series of bioassays were performed in which leaves of Col-0 wild-type and *aterf-1* plants were infiltrated with the *Pst* strain and disease symptoms developed on their surfaces were compared (Figure 6A, B and C). The number of leaves that displayed extensive chlorotic lesions was much higher in the *aterf-1* mutant than in the Col 0 (Figure 6B), and the Disease Index indicated a highly significant difference between both genotypes (Figure 6C). A different type of bioassay measured the *in planta* growth of the *Pst* bacteria at different times post-infection (Figure 6D); in accordance with the symptoms developed by the plant, bacterial growth was significantly higher in the *aterf-1* mutant. These results demonstrate that the *ATERF-1* gene product is required *in vivo* for a proper defence response to *Pst* infection.

### 7 Systematic identification of selected clones by microarrays

Next, we developed a microarray-based strategy to allow for systematic identification and quantification of clones selected by bio-panning. Clones shown in table 2 were easily isolated since they were present at high frequency in the bio-panned eluates, which contained as much as 8.6×10^7^ pfu (Figure 2A). However, the identification of less frequent but still valuable clones requires large-scale analysis. The isolation of individual pfu and the sequencing of their cDNA inserts is a time-consuming and expensive procedure when large number of clones needs to be analysed. On the other hand, although redundancy of a clone after bio-panning suggests affinity selection, a clone that was already very frequent in the initial library would be also expected at high frequency after a random selection. Therefore, the abundance of a clone before and after bio-panning should be compared to determine the enrichment yield after selection.

To this end, we used Arabidopsis, two-colour microarray probes to rapidly quantify the copy number of each cDNA insert before and after bio-panning. The inserts contained in the libraries (L) or in the bio-panned eluates (B, rounds 1 to 5) were PCR-amplified, labelled with Alexa 555 or 647 fluorochromes and hybridized to Quiagen AROS Version 3 microarrays. The signals from the T7LAtPa or T7LAtPs libraries were used as the reference colour channel and 4 replicates were performed for each B *versus* L comparison in order to provide error estimations for the statistical analysis. Normalized values from all the probes are provided in supplemental datasets S3–S5.

#### 7.1 Significance analysis of *P. aeruginosa* bio-panning

**Figure 7:**
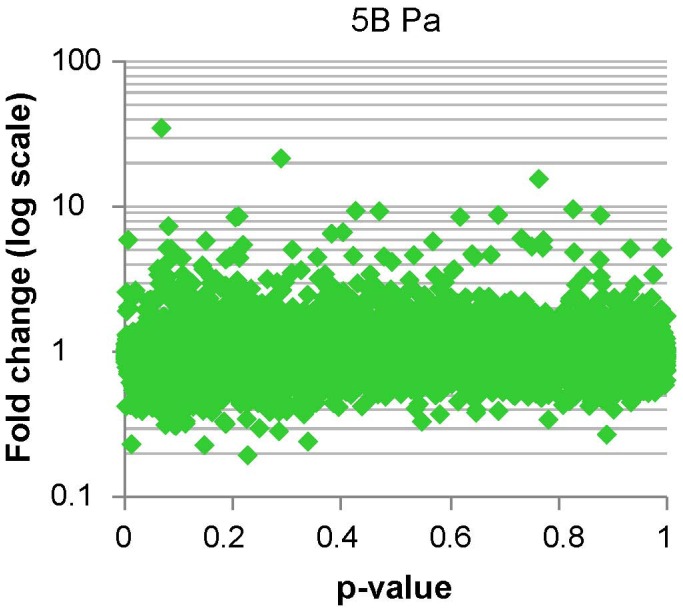
Microarray analysis of the *Pa* bio-panning, significance *vs.* fold-change plot. cDNA from the T7LAtPa library (L) and the clones selected after bio-panning (B) was hybridized to AROS microarrays. For each gene spotted in the microarray the B/L ratio and the statistical significance of B/L≠1 were determined. The plot represents the B/L ratio as a fold-change in the Y-axis (log values centred around 1) and the p-values for the statistical test in the X-axis. Genes with highest fold-change (maximum enrichment after bio-panning selection) and lowest p-value represent the best candidates as *Pseudomonas*-bound clones.

Significance analysis of microarrays was performed with GeneSpring software using the *t*-test against 0. This analysis provided p- and fold-change values for each gene spotted on the microarray, which are fully listed in S6 dataset. The dataset is represented as a plot in Figure 7. To select for genes with maximal fold-change after selection but significant p-values we used a cut-off plus multiple testing correction. Since stringency of multiple testing corrections depends on how many genes are tested, a first cut-off on fold-change (>|±1.45|) and p-value (<0.1) was used to reduce the number of tests, and 806 genes were pre-selected for correction (Table 3). From here genes with negative fold-change (genes that were selected against during the bio-panning) were removed and then False Discovery Rate (FDR) correction was applied to define a final set of 101 candidates with corrected p<0.05 (S7, S10).

**Table 3:** Microarray selection of three sets of 101 (*Pa*), 153 (*avr*) and 318 (*Pst*) genes with significant fold-change after bio-panning.

Bio-panned library	Bio-panning substrate	Bio-panning round	Genes with |FC|>1.45 and p<0.1	Genes that pass FDR correction
**T7LAtPa**	***Pa***	**5B**	806	**101**
**T7LAtPs**	***avr***	1B	121	31
		2B	488	122
		3B	10	9
		**1B U 2B U 3B**		**31 U 122 U 9 = 153**
	***Pst***	1B	531	171
		2B	374	142
		3B	24	17
		**1B U 2B U 3B**		**171 U 142 U 17 = 318**

FC Fold Change, FDR False Discovery Rate.

#### 7.2 Significance analysis of *P. syringae* bio-panning

**Figure 8:**
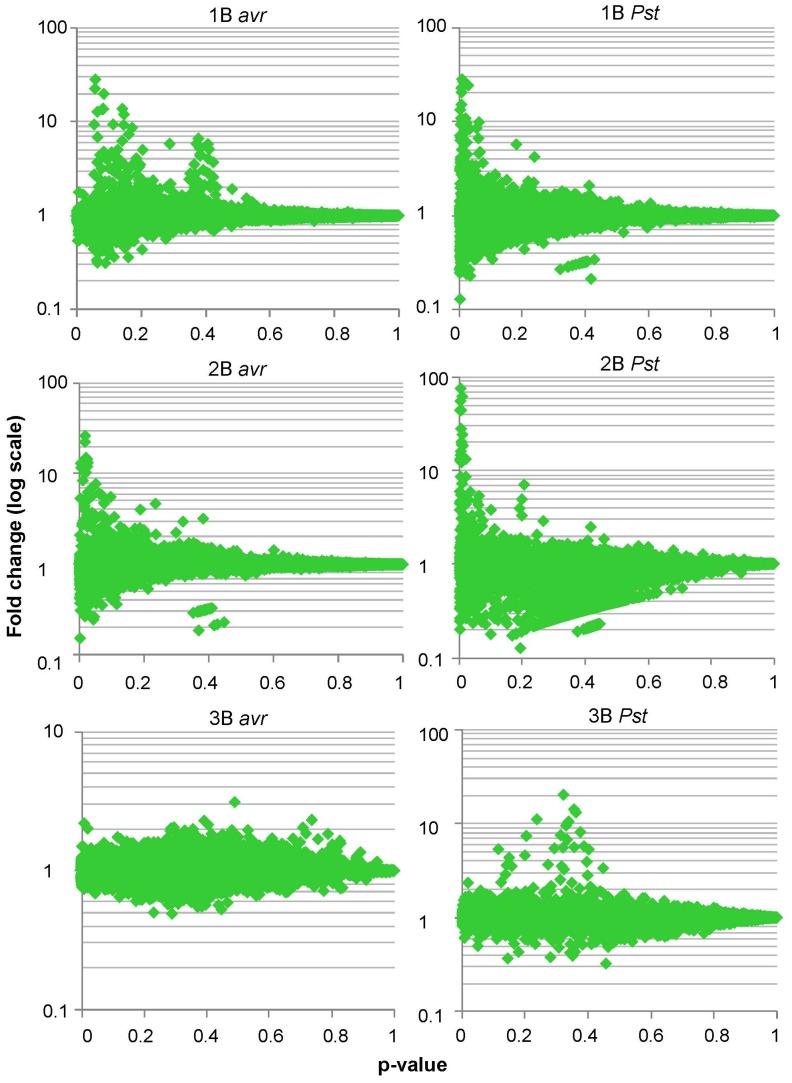
Microarray analysis shows significant fold-changes in rounds 1 and 2 of *Pst and Pst(avrRpt2) (avr)* bio-pannings. cDNA from the T7LAtPs library (L) and the clones selected after bio-panning rounds 1, 2 or 3 (1B, 2B or 3B) were hybridized to microarrays. For each gene spotted in the microarray the B/L ratio and the statistical significance of B/L≠1 were determined. The plot represents the B/L ratio as a fold-change in the Y-axis (log values centred around 1) and the p-values for the statistical test in the X-axis. Best candidates to be *Pseudomonas*-bound clones are represented by the spots with highest fold-change and lowest p-values, which are located on the top-left area of the graph.

The fold-change and p-value for each gene were calculated from the *t*-test as described for *P. aeruginosa* microarrays (full list in S6). In this case we observed that in rounds 1 and 2 the genes with highest fold-changes were concentrated around the lowest p-values, whereas in the 3^rd^ round fold-changes were more evenly distributed along the p-value axe (Figure 8). In addition, fold-changes in the 1^st^ and 2^nd^ rounds were overall higher than in the 3^rd^ round of selection. Thus, the best candidates (maximal fold-change with the minimal p-value) were already defined in rounds 1 and 2. Consequently, we delimited the final set of candidates taking into account microarray data generated during the 3 rounds of selection. The genes that passed the FDR correction in at least one round of bio-panning defined two sets of 153 and 318 genes from *Pst*(*avrRpt2*) and *Pst* strains respectively (Table 3 and S8, S9, S10).

#### 7.3. Overlapping of significant gene sets

**Figure 9:**
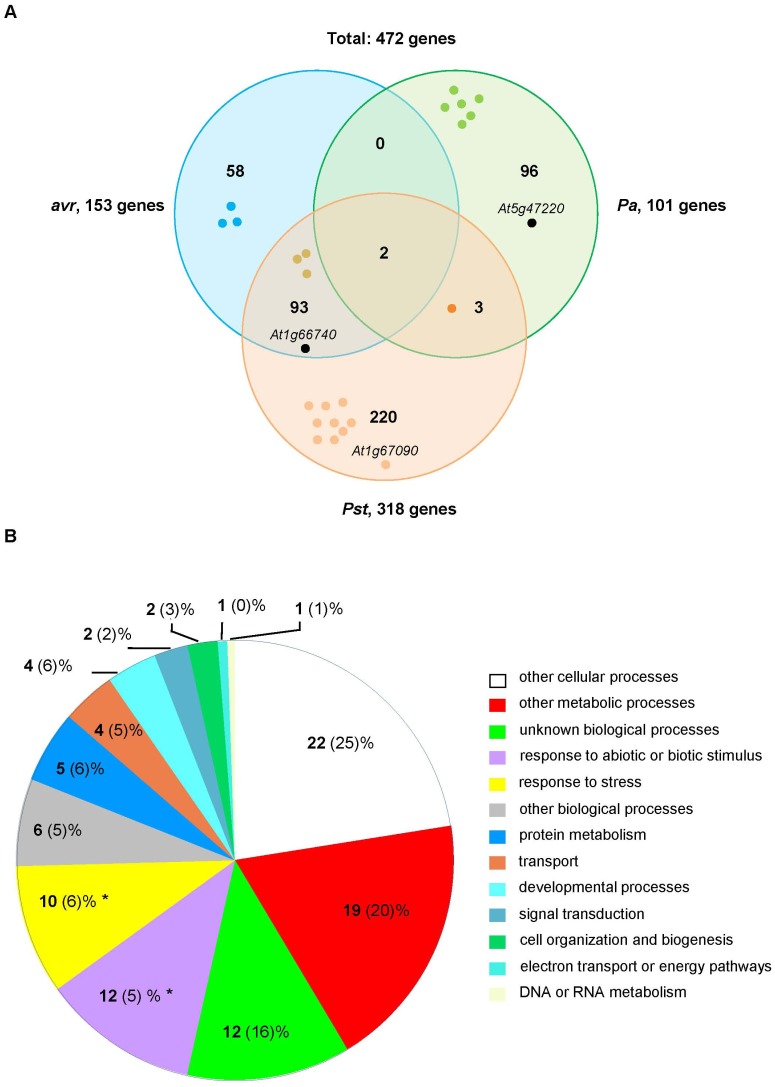
Overlapping and categorisation of the gene sets defined by significance analysis of microarray data. (**A**) The analysis identified 3 sets of genes: 101, 153 and 318 genes were selected after bio-panning against *Pa*, *Pst*(*avrRpt2*) (avr) and *Pst* strains respectively. The sets are represented as overlapping Venn diagrams and the number of shared genes is shown in the intersections. The union of the 3 sets produces a total of 472 genes as candidate MAMP/effector ligands. The positions of *At1g66740* (*AtSP7*), *At1g67090* (*RBCS1A*) and *At5g47220* (*ATERF-2*) are shown in each set. Gene products that produced immune interactions in PPIN-1 are represented by coloured dots. (**B**) Genes in the 472-gene set were annotated according to the GO categories for Biological Process and the percentage of annotations falling in each category (in bold) was compared to the percentage produced by the 26,303 genes spotted on the microarray (in brackets). Categories with significant (p<0.01) over-representation are shown by asterisks.

The 101-, 153- and 318-gene sets define three groups of phage clones for which copy number was significantly increased after bio-panning with *Pa*, *Pst*(*avrRpt2*) and *Pst* strains respectively. The union of the three sets contains 472 genes which represent potential MAMP/effector-interacting proteins. The overlapping among sets is shown in Figure 9A. As expected, the two strains of *P. syringae* shared a large number of genes (95), whereas *Pa* shared only 5 genes with the *Pst* strain, 2 of them common to *Pst*(*avrRpt2*). Three subsets of 96, 58 and 220 genes remained specific for *Pa*, *Pst*(*avrRpt2*) and *Pst* strains respectively. The genes in each subset are listed in S11–S16. We inspected the lists to identify in-frame clones already sequenced in table 2. Since the microarray probe for *ATERF-1* is located at the very N-terminus of the predicted protein, this probe does not cover the fragment that is present in the T7-ATERF-1 clone, and therefore the clone could not be identified by hybridisation to microarrays. However the lists includes *AtSP7* (*At1g66740*) as a top-represented gene (see complete list of fold-change values in S6) and *RBCS1A* (*At1g67090*). Both genes were identified by sequence analysis of the dominant clones rescued after bio-panning. Thus, our significance analysis confirmed in-frame proteins identified through the first approach to characterize *Pseudomonas*-bound clones. The 472-gene set also contained 23 targets (S17) that produced immune interactions in the Plant-Pathogen Immune Network-1 (PPIN-1) [18], including *RBCS1A*.

The 472 genes were grouped into broad functional categories based on the GO hierarchy. The percentage of genes falling in each category was compared between this set and the whole set of genes in the microarray (26,303 genes, Figure 8B). The 472 gene-set was significantly enriched in genes falling in the categories of response to abiotic/biotic stimulus and response to stress (12% and 10% in the 472-gene set compared to 5% and 6% in the microarray respectively). This analysis confirmed that the 472 genes are not a random selection from the microarray gene-set and include a significantly increased number of representatives for biological categories consistent with a role as immune targets.

### 8 Defence-related genes in the significant sets identified by microarays

Genes falling in the broad categories of response to biotic/abiotic stimulus or stress response were inspected to find the best candidates for MAMPs/effector targets. The genes annotated as defence-related in the fine GOslim classification were listed apart (Table 4). This list includes different molecular families involved in plant immunity, some of which are discussed next.

**Table 4:** Significant genes falling in the GO fine category of “defence response”.

Gene set	Gene ID	Sub set	1B FC	2B FC	3B FC	5B FC	Description
**101 ** ***Pa***	At1g09340	96	-	-	-	5.4	CRB CHLOROPLAST RNA BINDING protein, putative
	At3g04720	96	-	-	-	5.1	PR4 PATHOGENESIS-RELATED 4
	**At3g11010**	96	-	-	-	3.0	RLP34 Receptor like protein 34 (LRR-containing N-terminal domain, type 2)
	At5g47220	96	-	-	-	2.9	ATERF-2 ETHYLENE RESPONSE FACTOR 2
	At4g23670	96	-	-	-	2.5	Major latex protein-related
	At2g31880*	96	-	-	-	1.7	LRR transmembrane protein kinase, putative
**153 ** ***avr***	At1g24020	93	1.4	2.2	−1.2	-	MLP-like protein 423
	At3g28930	58	1.0	1.6	−1.3	-	AIG2 AVRRPT2-INDUCED GENE 2
	At1g63870*	58	−1.0	1.5	−1.1	-	Disease resistance protein (TIR-NBS-LRR class), putative
	At5g06320	58	−1.2	−1.0	1.5	-	NHL3 NDR1/HIN1-like 3
	At3g27850	93	−1.4	3.1	1.0	-	RPL12-C RIBOSOMAL PROTEIN
**318 ** ***Pst***	At1g24020	93	3.9	−1.1	−1.0	-	MLP-like protein 423
	At1g79210	220	2.5	−1.0	1.1	-	20S proteasome alpha subunit B
	At3g21220	220	2.1	−1.2	1.1	-	ATMKK5 MITOGEN-ACTIVATED PROTEIN KINASE KINASE 5
	At1g52660*	220	1.9	−1.4	1.4	-	Disease resistance protein, putative (NB-ARC, LRR type 3)
	At4g23810	220	1.9	−1.2	−1.0	-	WRKY53 transcription factor
	At2g15220	220	1.5	1.2	−1.0	-	Plant basic secretory protein (BSP) family protein
	At3g27850	93	1.5	−3.3	−1.5	-	RPL12-C RIBOSOMAL PROTEIN
	At5g53890*	220	1.0	1.9	1.3	-	LRR transmembrane protein kinase, putative
	At1g19610	220	−0.9	1.5	1.2	-	LCR78/PDF1.4 Low-MW-cysteine rich 78
	At2g43530	220	−2.5	3.8	2.0	-	Trypsin inhibitor, putative

FC Fold change, 1 to 5 rounds of bio-panning (1B to 5B). Predicted LRR-containing proteins are marked in bold, asterisks indicate immune-related baits tested by Muhktar *et al*.

The most represented family of proteins in Table 4 is the group of LRR receptors [37], with 5 representatives. From them, *At2g31880* has been previously reported as a flagellin-responsive gene. *At2g31880* expression is elicited by flagellin or *Pseudomonas* infection and activates defence responses through a signalling pathway that is repressed by *BIR1*, a negative regulator of *BAK1* [38], [39]. BAK1 and its partner FLS2 are themselves LRR-containing receptors that signal pathogen presence by interacting with flagellin [40], [41] but also with pathogen-derived effectors like AvrPto [42], [43]. Our data suggest that the mechanism by which *At2g31880* activates defence involves the binding between the encoded protein and a *Pseudomonas*-derived molecule, which could also be in the basis of *BIR1* inhibitory action.

The table also includes 2 genes encoding for nuclear proteins: *ATERF-2* (*At5g47220*) and *WRKY53* (*At4g23810*). *ATERF-2* is the closer homologe to *ATERF-1* and both transcription factors share extensive similarity within and outside the ERF domain [30], [32]. *WRKY53* is a positive regulator of basal resistance triggered by virulent pathogens/MAMPs and is tightly regulated at various levels, *i.e.*, its interaction with the MEKK1 kinase that signals pathogen infection [44], [45]. This mechanism is a short cut to directly activate WRKY53-controlled genes upon pathogen infection, which shows that transcription factors involved in plant defence have evolved several check-points to sense pathogen attack.

*AIG2* (*At3g28930*) and *NHL3* (*At5g06320*) are in the subset of genes that were specifically identified after bio-panning with *Pst*(*avrRpt2*) strain (58-gene subset). *AIG2* was one of the first genes isolated that exhibited *RPS2*- and *avrRpt2*-dependent transcript induction early after infection with *P. syringae* strains carrying *avrRpt2* [46]. Although its function remains unknown, the protein structure suggests that AIG2 can bind small ligands in a hydrophilic cavity as a part of its active site [47]. *NHL3* encodes for a plasma membrane protein responsive to *P. syringae* infection which has been proposed to function as an R receptor for Avr proteins [48].

In the Pst-specific group (220-gene subset) there are 8 genes previously annotated as defence-related. *At1g79210* encodes for an endopeptidase which forms part of the 26S proteasome complex and accumulates differentially in response to inoculation with *Pst* or *avrRpm1* strains [49]. *At3g21220* encodes for the AtMKK5 kinase. This protein has been shown able to interact directly with the HopF2 effector of *P. syringae* and interfere with MAMP-triggered immunity [50]–[52]. *At1g19610* and *At2g43530* are included in the defensin family of proteins [53]. Defensins bind strongly to microbial surfaces as a first step to exert they antimicrobial function [54], and so they are good candidates to be rescued by affinity bio-panning.

**Figure 10:**
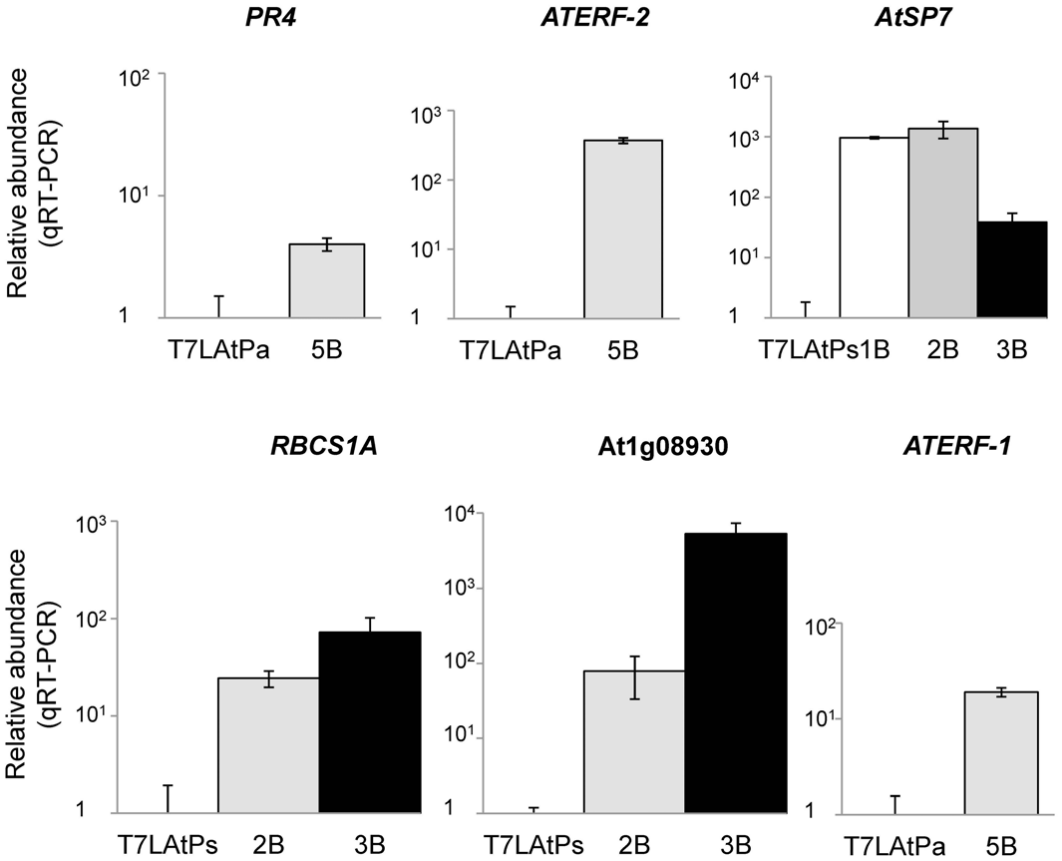
Quantification of microarray-selected candidates by qRT-PCR. Relative abundance of each gene was measured before selection in the libraries (T7LAtPa or T7LAtPs) and after bio-panning selection in the eluates (1 to 3 or 5B). Columns represent normalized values from 3 replicas respect to an internal sequence in the T7-Select10 3b vector. All the genes were over-represented after selection, with significant p-values.

Quantitative Real-Time PCR (qRT-PCR) was performed on a number of these genes to validate our microarray analysis (Figure 10). Defence-related and top-regulated genes selected from the significant subsets of *Pa* (*PR4* and *ATERF-2*), *Pst*(*avrRpt2*) (*AtSP7* and *At1g08930*) and *Pst* (*RBCS1A*) bio-panning analysis were tested, as well as *ATERF-1*. qRT-PCR confirmed a significant enrichment of these genes after bio-panning selection in all the cases.

## Discussion

Phage display is a powerful technique that allows for the expression of a large number of proteins on viral particles and their selection on the basis of their binding affinity for a ligand. In this study we used the technique to perform a high-throughput selection of Arabidopsis proteins with affinity for *Pseudomonas* bacteria and therefore with a putative role in natural plant-microbe interactions. Phage-display-based strategies have been shown before suitable for the selection of plant proteins with physiological ligands [55], [56], but to date no attempts to use them in a wide screening of plant immunity targets have been reported. To provide input for this selection we constructed two phage-displayed libraries covering different fractions of the plant immune transcriptome. To identify the output we took advantage of microarray development in Arabidopsis and coupled microarrays to phage display in a novel tool for research. This innovative approach stood for the quantification of all putative binders in a genome-wide scale and provided a significant list of candidate targets for MAMPs and virulence effectors. Clone identification is the last and less efficient step of phage display, and only recently has been addressed by using high-throughput technologies [21].

Three different plant-pathosystems were used as a cDNA source for the construction of the libraries. In the Arabidopsis *vs. Pst* pathosystem, infection with the bacterium results in a compatible interaction and induces de transcription of a large number of plant genes [31], [57]. Infection with the *Pst*(*avrRpt2*) strain elicits the HR response in hosts that recognize the AvrRpt2 protein and results in incompatible interaction. The use of *P. aeruginosa* in the third pathosystem has additional interest since this bacterium is an opportunistic pathogen of humans with an extended range of hosts [58]. *P. aeruginosa* PA14 strain is a hypervirulent isolate that produces pyonacin and other factors of virulence both for mammalian and plant hosts [24], [27], [28]. The three strains share MAMPs common to most Gram-negative bacteria, like LPS or flagellin, and secrete virulence factors some of which are able to subvert host defences activated by MAMPs. Recently, it was shown that *P. aeruginosa* is able to evade immune recognition of flagellin through a similar mechanism in mammals and plants [59]. Unlike *P. syringae*, this bacterium has not evolved to be nutritionally dependent of a plant host [26]. Thus, these 3 pathosystems represent different degrees in the specificity of the plant-microbe interaction during which the host response might involve a broad range of molecules that recognize, signal and neutralise MAMPs and/or virulence effectors.

As the vector for phage display expression we used T7, since display in the lytic phage can produce libraries of greater diversity than M13. According to our estimations in Table 1 and S1 the T7LAtPs library constructed in this study represents virtually the entire AtORFeome. Our determination of phage numbers in the primary library was based on pfu-counting; however quantification by real-time PCR provides estimations 5 to 10 times higher [21]. Thus, the actual coverage of these libraries might be higher than reported here. Since the average size of cloned fragments ranges from 0.2 to 1.5 or 2.2 kb, full-length cDNAs should be represented to some extent, although N-terminal domains are under-represented as observed from Table 2. The screening of 6.3×10^9^ pfu that we performed for each pan represents every possible cDNA in the most complex, T7LatPs library with a multiplicity of 100. This allows sufficient sequence representation to find rare cDNAs during selection.

The next step in our strategy involved the selection of affinity clones by bio-panning against a living substrate. Living cells like zoospores or cultured cell lines [60], [61] have been bio-panned against peptide libraries. We used bacterial cells that were pre-incubated with plants to ensure the expression of effectors induced only upon host contact. To follow the enrichment in affinity clones we monitored the titres of eluates during successive rounds of selection (Figure 1). When bio-panning high-affinity antibodies titres usually increase up to a maximum that indicates the round from which eluted clones should be analysed. The selection and amplification of the clones bound in the first round results in increasing titres in later rounds, but once the eluates are saturated for affinity-binding clones titres plateau since no further selection happens and clones amplify similarly. This is not necessary observed when selecting for low-affinity interactions where clones compete weakly for binding to the substrate. In our case, the iteration of pannings against bacteria but not against the agarose control resulted in titres that were increased 10^2^–10^3^ times respect to the first round. This is a significant increase, similar to values reported for antibody libraries. However, maximum titres of rounds 5^th^ and 3^rd^ did not remain stationary, suggesting that bio-panning was not driven to saturation. The point of maximum enrichment for affinity-binding clones depends on the complexity of the library and the requirements of each displayed protein *vs.* substrate interaction, since phage selected in one round compete to each other in different proportion during the next round. In our bio-panning we introduced an additional level of complexity because bacterial cells can display many different ligands for selection at the same time. The analysis of the dominant clones isolated from rounds with titration peaks (Figure 2 and Table 3) was relevant since the T7-ATERF-1 clone confirmed its binding properties in the competition assay. However, the isolation of a clone at high frequency in a particular round of bio-panning does not imply enrichment during selection. Microarrays allow for the quantification of the differences in the copy number of each clone during selection (B *vs.* L fold change), which can provide an absolute measurement for enrichment. In our study, B *vs.* L fold-changes, were monitored across the first 3 rounds of bio-panning with T7LAtPs library, and this provided additional data about the kinetics of selection. From significance *vs.* fold-change plots in Figure 7 the clones with lowest *p*-value and highest fold-change appeared in the first and second rounds of selection; therefore we included these data in the significance analysis (Table 3).

The S6 dataset summarizes the most relevant information produced by our genome-wide analysis: for each gene spotted in the microarray a fold-change value is provided plus its associated significance level. We choose significance analysis of microarrays as a supervised statistical procedure to define the best candidates, but a variety of methods are available for microarray selection that can be applied to our supplemental data. The significance analysis defined a total of 472 genes with significant fold-changes (Table 3), distributed in overlapping sets. The 101 gene-set (*Pa*) was selected from a different input (T7LAtPa library) and the low overlapping with the other two sets might be due to the lower coverage of this library. However, gene sets arising from *Pst* and *Pst*(*avrRpt2*) bio-pannings are comparable since both were selected from the same input (T7LAtPs). Thus the high overlapping between them (Figure 9A, 95 shared genes) likely reflects common targets for the two *P. syringae* strains. Mining of data in S10 might provide additional information about the specificity of the interaction with the three bacterial strains tested in his study, although interpretation about the biological relevance of these interactions during *in planta* defence response should be cautious. Overall, the 472-gene set represents a microarray selection of candidate genes based on the microbe-binding properties of their phage-displayed proteins. Further validation of individual candidates picked from the set is necessary to confirm their importance as immune targets.

A global study of the categories represented in the 472-gene set indicated over-representation of biological processes that are consistent with a role of the selected genes as putative targets for MAMPs or virulence effectors (Figure 9B). In addition, the comparison with previously published, transcript profiling datasets [31], [57] revealed that at least 120 genes in this set are also regulated at the transcriptional level during the *in vivo* response of the plant to bacteria (S17). A finer inspection through GO hierarchy detected 19 defence-related genes with very different molecular functions, discussed in detail in section 8. At least one of them (*At3g21220*) encodes for an *in vivo*-validated target of a bacterial effectors. Moreover, the 472-gene set contains 23 immune targets (S18) confirmed by protein interactome analysis [18] which are also very diverse in their molecular roles. NB-LRR disease resistance proteins are represented (*At1g52660*), but also metabolic enzymes validated by our sequence analysis (*At1g67090*) and transcription factors like SNZ (*At2g39250*), in the family of AP2-ERF DNA-binding proteins, or AtTCP15 (*At1g69690*). The latter was able to interact with several groups of Avr and Hop effectors from *Pseudomonas syringae* used as baits for the PPIN-1 map.

These results demonstrated that microarray hybridization plus significance analysis provide an efficient method for wide identification of putative targets. Since hybridisation does not pre-require isolation of clones, this approach is fast and produces information for many genes. However, clone isolation facilitates frame analysis and further characterization of the binding properties of selected protein. We used the T7-ATERF-1 clone isolated from bio-panned eluates to validate our selection strategy and investigated the role of ATERF-1 during plant-defence. The binding of T7-ATERF-1 to the 3 strains of *Pseudomonas* was compared and we found that, in addition to *Pa*, *Pst* strains also produced significant enrichments in recovered phage, whereas a Gram (+) bacterium did not (Figure 3). This suggested that the bacterial ligand for ATERF-1 is a molecule present in the 3 strains of *Pseudomonas* rather than a strain-specific virulence factor. In accordance, the nuclear translocation of *GFP-ATERF-1* protein was promoted equally by the 3 strains (Figure 4). Inoculation with heat-killed bacteria induced the same translocation effect, whereas a mock solution without bacteria failed to change subcellular location of the protein. Thus, translocation from the nucleus does not require the signals produced by living bacteria in the plant apoplast, and is not induced by wounding signals associated to inoculation. Although our results do not imply that the binding of a bacterial ligand to ATERF-1 causes the exportation from the nucleus, the two phenomena share common features. Bacterial effectors like PopP2 can interact with host proteins to modulate their nuclear localisation [35], [62].

In an effort to go deeper into the physiological role of ATERF-1, we assessed the resistance of an *aterf-1* mutant against *P. syringae* infection (Figure 6). The sensitive phenotype indicates that *ATERF-1* is required for a proper immune response and other defence mechanisms induced during natural response to the bacterium are not able to compensate for its effect. The ERF family of transcription factors is very redundant and loss-of-function mutants do not usually exhibit susceptible phenotypes [63]. In contrast to other members of the family [64]–[66], the expression of *ATERF-1* is induced not only by ethylene, but also by flg22 treatment and *Pst* infection [31], [57]. However, the transcriptional response to *Pst* is stronger than that to flg22 or ethylene alone [31]. According to these authors, this would indicate that ethylene basal response is insufficient to prevent *Pst* pathogenesis or that *Pst* is able to block ethylene signalling or responses downstream *ATERF-1* induction. Considering our results, it is tempting to speculate that a *Pseudomonas* ligand can be internalized into the nucleus where interacts with ATERF-1 to release transcriptional regulation by this factor. The finding that ATERF-1 activity is both sensitive to ethylene and pathogen-derived molecules suggests that the long-distance control of defence response by hormones and direct sensing of pathogen molecules can be integrated through the same transcription factor. Although classically transcription factors have not been considered as direct binders of the microbe-derived molecules that trigger immunity, there are recent examples of nuclear host proteins which are able to interact with bacterial factors [35], [67]. The RRS1-R protein from *A. thaliana* interacts with the *Ralstonia solanacearum* effector protein PopP2 in the nucleus of living plant cells [68]. RRS1-R is composed of two differentiated domains, with a DNA-binding motif which is characteristic of the Zin-finger class or WKRY transcription factors and a Toll/Interleukin receptor (TIR)-NBS-LRR-like domain for pathogen sensing. Similarly, *ATERF-1* (*At4g17500*) is composed of a C-terminal domain with homology to the ERF-1 family of transcription factors plus an N-terminal extension which is not present in any other member of the family.

The PPIN-1 interactome map revealed that transcriptional regulators represent the most enriched category in the subgroup of 165 putative effector targets [18]. The identification of a variety of immune targets as a result of our microarray analysis underscores the possibility that pathogen-sensing is a capacity retained in very different families of proteins that are involved in plant defence, from surface receptors to transcriptional regulators. Typical R proteins have a modular structure composed of different terminal domains in addition to their central NB-LRR region. This structure facilitates a tight regulation of their activity, which is accomplished by intramolecular interactions between the various domains [15]. The acquisition of pathogen-sensing domains during the evolution of structurally unrelated proteins would facilitate the integration of defence responses in the complex immune system of the plant.

## Materials and Methods

### Microbial strains

*Pseudomonas aeuriginosa* PA14 strain is a hyper-virulent isolate that was kindly provided by Prof. F. Ausubel (Massachusetts General Hospital, Boston, USA). *Pseudomonas syringae pv. tomato* DC3000 strain (*Pst*; wild-type, Rif^r^) and the *avrRpt2* strain containing the pV288 plasmid [29] were generous gifts from Dr. Jens Boch (Martin Luther Universitat, Halle, Germany). *Enterococcus faecalis* was provided through the CECT Spanish Collection of Culture Types. *Escherichia coli* DH5*α* (F^−^ recA Δ*lacU169*(*π80 lacZ*Δ*M15*) *endA hsdR gyrA*) was obtained from Dr. F. Fierro (Universidad de Leon, Leon, Spain) and BLT5103 and BL21 (F^−^ *ompT gal [dcm][lon] hsdS*_B_) strains from Novagen. *Agrobacterium tumefaciens* C58C1 strains containing the pGV2260 or the pCH32 plasmid are described in [69]. Unless otherwise noted, bacteria were grown in LB medium supplemented with ampicillin (100 µg/ml), kanamycin, rifampicin or gentamicin (50 µg/ml) when appropriate.

### Plant materials

*Arabidopsis thaliana* accession Columbia-0 (Col-0) is the genetic background used in this work. Arabidopsis seeds were surface-sterilized in 20% bleach and 0.05% Tween-20 for 90 s and washed five times in sterile water before sowing. Seeds were stratified for 3 days at 4°C and then sown on Petri dishes containing Murashige and Skoog medium (MS basal salts, 2–3% (w/v) glucose, 0.6% (w/v) agar pH 5.7). Plates were sealed and incubated in a controlled environment growth chamber. Seven- to ten-day-old seedlings were transferred to individual test-tubes containing 5 ml of liquid MS or sown on pots containing a sterile mixture 3∶1 soil-vermiculite and grown in the greenhouse with 16-h light/8-h dark photoperiod. Plants used for the bioassay with *P. syringae* were sown in autoclaved sand and grown in a controlled-environment chamber at 21°C, 70% relative humidity and 200 µM×m^2^/s of cool white fluorescence illumination (10-h light/14-h dark). After 10 days, germinated seedlings were transferred individually to 60-ml pots containing sterile soil-sand mixture (12∶5 v/v) [70]. *Nicotiana benthamiana* plants were grown in the greenhouse at 22°C and 16-h light/8-h dark cycle.

### Infection of plants with bacterial strains

For infection with *Pa*, 25-day-old plants grown in liquid MS were inoculated with OD_600_ = 0.02 as previously described [28]. Infected plants were incubated into a growth chamber at 30°C and 90 rpm, under long-day light conditions. For infection with *P. syringae* 4-week-old plants growing in pots were infected with OD_600_ = 0.002 of bacteria by using the vacuum infiltration procedure [71]. For *aterf-1* bioassays, series of eight 5-week-old plants were inoculated with OD_600_ = 0.0005 from an overnight culture (resuspended in 10 mM MgSO_4_). Bacteria were introduced in 4–5 rossette leaves per plant by pressure infiltration [69]. At 3 and 4 days post inoculation (dpi), leaves were classified by similar developmental state and disease symptom severity was scored using a scale consisting of three classes of lesions: 1 (0–10% of the leaf surface with necrotic lesions), 2 (10–50%), 3 (>50%). Disease index (DI) was calculated for each plant using the three-grade scale according to the formula: DI = (0*n*_1_+1*n*_2_+2*n*_3_)/*N*_t_ where *n*_1_–*n*_3_ is the number of leaves in the indicated class and *N*_t_ is the total number of leaves collected from one plant. To determine bacterial concentration in infected leaves, series of 8 plants per genotype and time point were inoculated in rosette leaves as before and the most developed leaves were collected to quantify bacterial titre by homogenizing 2 leaf discs (6 mm diameter) per plant in 400 µl of 10 mM MgSO_4_. Serial dilutions of this homogenate were plated on selective KB medium (2% Protease peptone, 0.15% MgSO4-7H_2_O, 0.2% KH_2_PO_4_, 1% Glycerol, 1.2% Agar and 25 µg/µl Rifampicin) and incubated for 48 h at room temperature before bacterial colonies were counted.

Transgenic *N. benthamiana* plants expressing *RFP:H2B* and the *35S:GFP-ATERF-1* transient-expression construct were infected with OD_600_ = 0.02 of *Pseudomonas* bacteria 3–4 days post-agroinfiltration. Bacterial cultures were centrifuged, resuspended in 10 mM MgSO_4_ and injected with a syringe into the leaves. Boiled bacteria were heated 10 min at 100°C. After the infection plants were incubated 3 hours into a growth chamber and observed with a confocal microscope.

### Construction of T7 -phage-displayed libraries from Arabidopsis cDNA

Plants were infected as described above and frozen in liquid nitrogen at different times post-inoculation (1 h, 3 h, 24 h, 48 h and 3–4 dpi to construct T7LAtPa library and 24 h, 48 h, 3 and 7 dpi for T7LAtPs libraries). For each time point, highly purified, total RNA was isolated from 5 g of frozen plants after homogenization with a micro-dismembrator (Braunn) as described previously [72]. mRNA was isolated from 400 µg of pooled RNAs to represent the transcriptional response of the plant during a time-course infection. The cDNA was synthesized from 4 µg of mRNA using OrientExpress cDNA Synthesis Kit (Novagen) and 2 µg of T_18_V_3_N to prime synthesis. A mix 2∶1 of MMLV RT (Novagen) and SuperScript III (Invitrogen) was used to synthesize first strand cDNA. Second strand synthesis and end modification were performed as recommended in the manual. End-modified cDNAs were fractionated by gel filtration using the Mini Column Fractionation Kit (Novagen) and higher molecular weight fractions were used for ligation intoT7Select10-3b vector *Eco*RI/*Hin*dIII arms. Different ligations were performed and independently packaged to achieve optimal vector∶insert ratios. Each packaging reaction yielded a different sub-library that was analyzed in order to determine the percentage and the size of the cloned inserts. Final libraries were generated by combining the most representative sub-libraries and scaling the packaging process up.

To calculate the number of primary recombinants, dilutions of the packaging reactions were mixed with *E. coli* BLT5403 and plated on LB+ampicillin plates as described in the T7-Select System Manual. After incubation at 37°C lysis plaques were counted to calculate phage titres, defined as pfu per unit volume. To determine the percentage of cDNA inserts cloned into the T7Select10-3b vector lysis plaques were transferred to a PCR mix and amplified with PIAG01 (5′ AGATTATCGCTAAGTACGC 3′) and T7ID (5′ GCAAGC(T)_18_ 3′) primers; a minimum of 271 (T7LAtPa) or 358 (T7LAtPs) pfu were analysed by this method during the construction of the sub-libraries. Amplification, storage of the libraries and related procedures were performed as recommended by Novagen.

### Bio-panning selection

*Pseudomonas* bacteria were grown to saturation in liquid LB and inoculated (OD_600_ = 0.16) into test tubes containing 10-days-old plants grown in 5 ml of liquid MS. Plants and bacteria were incubated together for 90 min without shaking at 30°C for *P. aeruginosa* or 25°C for *P. syringae*. Bacteria from 5 plants were recovered by gently rubbing the roots with a 1-ml tip containing MS. MS was then filtered through sterile Whatman paper to remove plant tissues. Bacteria were centrifuged and resuspended in 0.5 ml of the filtered MS before mixing them with 0.5 ml of LB containing 6.3×10^9^ pfu of recombinant phage. Bio-panning was performed for 30 min in 1.5-ml microtubes within a hybridization oven at 25–30°C and 70 rpm. After this time, bacteria were centrifuged 1 min at 13.200 rpm and rinsed by vortexing 1 min in 1 ml TBST (Tris–buffered saline, Tween 0.005%) a total of 5 times. For elution, the bacterial pellet was resuspended by pipetting 3–5 times in 200 µl of elution buffer (10 mM Tris pH 7, SDS 1%). 100 µl of this solution were amplified immediately for the next bio-panning round in 50-ml cultures of *E.coli* BLT5403 and 100 µl were preserved in 1 ml of saline mixture (0.5 M NaCl) for titrating and further analyzes.

For competitive bio-panning different input mixtures of T7-ATERF-1 or T7-ATUBA1 clones and T7-C1 control phage were prepared to a final concentration of 6.3×10^9^ pfu/ml and panned under the same conditions described above. Input and eluted mixtures were simultaneously titred and replica-analyzed by PCR of 24 to 96 clones from each titration series. The inserts contained in the clones were amplified using PIAG01 and PIAG02 (5′ ATAGTTCCTCCTTTCAGC 3′) primers to yield a 200-bp band for T7-C1 and a 600-bp or 550-bp band for T7-ATERF-1 or T7-ATUBA1 clones, respectively. 2–3 independent pans were performed from each input mixture to provide the final error estimations. Non-specific binding controls were performed by replacing bacteria with an agarose solution at similar OD_600_. For the LPS-binding assay, purified LPS from *P. aeruginosa* serotype 10^22^ (SIGMA) was coupled to agarose using ABH (p-Azidodenzoyl hydrazide, Thermo Scientific) as a cross-linker. LPS was cross-linked to a final concentration of 2.5 mM, which simulates a living cell. Agarose-coupled LPS was used as the substrate for competitive bio-panning experiments with the T7-ATERF1:T7-C1 input mixtures instead of *Pseudomonas* living cells. Bio-panning with rATERF-1 or rLACZ proteins was carried out by resuspending *Pst* cell pellet in 500 µl of 0.1× Protein Elution Buffer, which contained 10 µg of freshly obtained, recombinant protein. After incubation at 25°C during 30 min to block ATERF-1-binding sites, cells were pelleted and resuspended in the phage mixture. Subsequent bio-panning was performed upon addition of 10 µg of recombinant protein to the cell/phage mixture, maintaining equivalent conditions of salinity (30 mM NaCl present in the 1× Protein Elution Buffer) and protein concentration (10 µg/ml) for incubations with rATERF-1 or rLacZ.

### PCR analysis, sequencing and clone rescue

To monitor the enrichment in particular clones during bio-panning a specific PCR-procedure was used. Phage eluted after each round of selection were amplified to >10^9^ pfu/ml and used to prepare high quality phage DNA by precipitation with 50% PEG 8000 (T7 Select-system Manual) and successive phenol extractions to remove phage capsid proteins. The cDNA amplicons contained in this clone mixture were amplified by PCR [94°C 2 min, 30×(94°C 30 s, 48°C 30 s, 68°C 3 min) 68°C 5 min] with PIAG01 and T7ID primers and Expand high fidelity polymerase (ROCHE). For individual-pfu analysis phage plaques were directly tipped into the PCR mix and analyzed with Taq (Invitrogen). For sequencing the PCR products were sub-cloned into the pCR2.1 vector (Invitrogen) and submitted to SISTEMAS GENOMICOS as DNA or colony plates when a large number of sequences were required. T7-ATERF-1 and T7-ATUBA1 clones rescued from the plates were amplified from eluates of *Pa* 5B round or *Pst* 3B round. Lysis plaques were picked with a sterile tip and kept in 100 µl of Phage Extraction Buffer (PEB, 20 mM Tris-HCl pH 8.0, 100 mM NaCl, 6 mM MgSO_4_). Aliquots of 1.5 µl were used for PCR amplification with PIAG01 as the common 5′ primer and T7-ATERF-1Rvs (5′ TCAACAACCTCGCACTTCAC 3′) or T7-ATUBA1Rvs (5′ AACGTAGGGCAGATGCAGAG 3′) as specific 3′ primers. Positive clones were amplified from PEB in 2 ml of BLT5403 (OD_600_ = 1) until cell lysis was observed and centrifuged at 8000 g for 10 minutes. The supernatant was re-amplified in 20 ml of bacterial culture under similar conditions. Lysates were filtered through a cellulose acetate filter (0.45 µm) and kept in 0.5 M NaCl.

### Labelling of cDNA inserts for microarray hybridization

To generate microarray probes 100 ng of highly-purified phage DNA was amplified by PCR with the PIAG01 and T7ID primers. PCR was performed in 100 µl with 1.5 units of Expand High Fidelity Plus PCR (ROCHE) under the following cycles: 94°C 2 min, 30×(94°C 30 s, 48°C 30 s, 68°C 3 min) 68°C 5 min. The product of 5 PCR reactions was purified before labelling with QUIAquick PCR purification columns (QUIAGEN). Labelling was performed with Alexa 555 or 647 fluorochromes using 4 µg of PCR-amplified DNA and the BioPrime® Plus Array CGH Indirect Genomic Labelling System (Invitrogen).

### Microarray hybridization and analysis

Arabidopsis Genome Oligo Set (AROS) Version 3 microarrays were provided by The University of Arizona. Microarrays were re-hydrated according to the manufacturer's instructions (http://ag.arizona.edu/microarray/methods.html) prior to hybridization. Microarrays were pre-hybridized and washed as previously described [73], and hybridized with 60 pmoles of each DNA-incorporated dye after denaturing in 90 ml of hybridization solution (50% formamide, 3× SSC, 1% SDS, 5% Denhard's reagent, 5% dextransulfate). Hybridization was carried out overnight at 42°C in a Corning hybridization chamber immersed in a water bath. Labelled samples were co-hybridized on the same microarray as follows: T7LAtPa library (L) *versus* 5^th^ bio-panning (5B) round with *Pa* (5BPa*vs*L), T7LAtPs versus 3^rd^, 2^nd^ or 1^rst^ bio-panning round (3B, 2B or 1B) with *Pst* (3BPs*vs*L, 2BPs*vs*L or 1BPs*vs*L), T7LAPs versus 3^rd^, 2^nd^ or 1^rst^ bio-panning round with *Pst*(*avrRpt2*) (3BAv*vs*L, 2BAv*vs*L or 1BAv*vs*L). For each B*vs*L comparison 4 replica microarrays were hybridized swapping the dies of L and B labelled DNAs. Spot signals were captured using a confocal GeneChip scanner (BIO-RAD) and the Vers-Array software. Captured data were lowess-normalised, averaged and statistically analyzed following the workflow for two-colour experiments implemented in the GeneSpring GX Software (Agilent), with L signals used as the control channel for normalization. For significance analysis the GeneSpring *t*-test was used to determinate if the expression values (log B/L centred around 0) for each gene were significantly different from 0. The p-value from the *t*-statistics was computed asymptotically with n = 100 permutations. Filtering with volcano plots was performed on p-values (p<0.1) and absolute fold change (>|±1.45|) of 5B*vs*L for *Pa* bio-panning or 1BvsL, 2BvsL and 3BvsL for *Pst*(*avrRpt2*) and *Pst* bio-pannigs. Corrected p-values were calculated with the Benjamini and Hochberg FDR correction on the minimum set of genes selected after each comparison, ie. 276-gene set for *Pa*; 31, 122 or 9-gene sets (1B, 2B and 3B respectively) for *Pst*(*avrRpt2*); and 171, 142 or 17-gene sets (1B, 2B and 3B respectively) for *Pst*. Supplementary data are shown as exported from GeneSpring GX gene-lists under the corresponding experiment interpretation. Functional categorization was performed with the Gene Ontology tool at TAIR (http://www.arabidopsis.org/tools/bulk/go). Comparisons with the PPIN-1 were performed by importing into GeneSpring a list of 841 genes from *A. thaliana* that produced immune interactions as described in [18]. For comparisons with the transcriptionally regulated genes reported by Truman *et al.* [57] or Thylmony *et al* [31] two lists of respectively 3,718 or 2,800 differentially regulated genes were imported and compared in Excel.

### qRT-PCR analysis

High quality phage DNA from the eluates recovered after bio-panning selection was subjected to quantitative PCR in one StepOnePlus RT-PCR thermocycler (Applied Biosystems) by using the following primers for target genes: qAtSP7FW1 (5′ TGTTGGCAACTACCGCTTTG 3′), qAtSP7RV1 (5′ AGGCTCTTCCTTGAGTTGCTC 3′), qERD6FW1 (5′ TCTGCAATGGGTTTGAGTGC 3′), qERD6RV1 (5′ ACACAATGTGACCCAAGACG 3′), qRCBS1AFW1 (5′ AGCTTCACCGGTTAATTTCCC 3′), qRCBS1ARV1 (5′ CGCAAACCGGAAAACAAACG 3′), PR4FW1 (5′ GGCCATCTCATTGTTGACTACC 3′), qPR4RV1 (5′ CAATGGCCGAAACAAGCATG 3′), qATERF2FW1 (5′ TGGTGATGAGACACGTGTTG 3′), qATERF2RV1 (5′ CACGGGAACACTTTTACTTGGTG 3′), qATERF1FW1 (5′ AGCTAGGGTTTGGTTAGGAACG 3′), qATERF1RV1 (5′ ATTCAACAAAGCGCGGGAAC 3′). Primers qT7S10FW1 (5′ TGTTAAGCTGCGTGACTTGG 3′) and qT7S10RV1 (5′ TCACACCTGACTGGAATACGAC 3′) were used to amplify the internal control. Amplification was monitored by using the Brilliant® SYBR® Green QPCR MasterMix (Stratagene), following the manufacturer's instructions. The thermal profile followed in these experiments was 50°C for 2 min, 95°C for 10 min, followed by 40 cycles of 95°C for 15 s and 60°C for 1 min and finally 50 s at 72°C. Quantification was repeated three times to get representative values for the slope of the standard curves and the standard deviation. The concentration of samples was calculated with the StepOnePlus RT-PCR software, which created threshold cycle values (Ct) and extrapolated relative levels of PCR product from the standard curve. The levels of PCR product for each tested gene were normalized against the internal control in the T7-Selelect 103B vector sequence. Bar graphs show normalized levels relative to the calibrator (PCR product detected in the libraries, before bio-panning selection) as log10 of the 2-ΔΔCt value.

### Isolation of the *aterf-1* insertional mutant

Seeds of the *aterf-1* mutant (SALK_036267) were obtained from NASC. Plants homozygous for the T-DNA insertion were confirmed by PCR amplification using a T-DNA specific left border primer (T-DNA SALK Lba_1_, 5′ TGGTTCACGTAGTGGGCCATCG 3′) and a forward *ATERF-1*-specific primer (ATERF-1-LPb, 5′ CGTCCATCTCATCCGAAAAT 3′). The amplicon confirmed the presence of the T-DNA insertion. The wild type *ATERF-1* locus was identified by PCR amplification using *ATERF-1* specific primers (ATERF-1-LPb and ATERF-1-RPb, 5′ CGTCGGAAGACGAAGAAGAC 3′). Loss-of-function was checked by Real Time-PCR using *ACTIN8* as endogenous control for expression levels and the *ATERF1* specific primers (qATERF1FW1 and qATERF1RV1) for the amplification.

### GFP fusions and *Agrobacterium* infiltration analysis

*ATERF-1* (*At4g17500*) full length cDNA was obtained from ABRC (U16643) and translationally fused to the C-terminal region of the GFP gene in the pMDC43 vector [74] by using the GATEWAY technology (Invitrogen). The resulting construct (*35S:GFP-ATERF-1*) was transformed into *A. tumefaciens* C58C1 strain carrying the pGV2260 plasmid, which delivered it into the leaf cells of *RFP:H2B* transgenic *Nicotiana benthamiana*. This transgenic line constitutively expresses red fluorescent protein (RFP) that is targeted to nuclei [75]. Simultaneous agro-infiltration with the pGV2260 and the pCH32-carrying strains was performed as described [76] to avoid gene silencing. Basically, *Agrobacterium* cells were resuspended in 10 mM MgCl_2_, 10 mM MES pH 5.6 and 200 µM acetosyringone and infiltrated in tobacco leaves by using a 10 ml-syringe. After infiltration plants were maintained for 3–4 days in a growth chamber before exposure to *Pseudomonas* strains.

### Fluorescence microscopy

The fluorescence photographs were taken with a Leica SP5 confocal microscope and Bio-Rad Radiance 2100 laser scanning confocal imaging system (LaserSharp v.5 Image software). For GFP and RFP detection, the excitation source was an argon ion laser at 488 nm and detection filters between 426–481 nm and 498–554 nm, respectively. Quantification of fluorescence was performed by using the Stack Profile tool of the Leica Application Suite AF Lite software (Version 2.3.5).

### Generation of transgenic Arabidopsis plants

Arabidopsis Col-0 plants were transformed with OD_600_ = 0.8 of *Agrobacterium tumefaciens* carrying the construct *35S:GPF-ATERF1* by the floral dip method. Basically, 200 ml of the bacterial culture were grown at 28°C and 250 rpm, centrifuged 10 minutes at 7000 rpm and resuspended into 200 ml of transformation solution (MS medium, 5% sucrose, 4.4 µl BAP (500 µg/µl), 60 µl Silwet L-77). Flowers were dipped into the transformation solution and vacuum-infiltrated twice during 30 sec. Seeds from transformed plants were harvested and plated on Hygromicin selective MS medium (1% Sucrose, 0.3% Phytagel and 40 µg/ml of Hygromicin) to identify T1 transgenic plants. Approximately 100 of T2 seeds were plated on hygromicin-containing MS and transgenic lines with a 3∶1 (resistant∶sensitive) segregation ratio were selected. T3 progeny, homozygous for hygromicin resistance, was used for further studies. Gain-of-function was checked by Real Time-PCR using *ACTIN8* as endogenous control for expression levels and the *ATERF1* specific primers (qATERF1FW1 and qATERF1RV1) for the amplification.

### Subcellular fractionation and Western Blot analysis

Rosette leaves from 4-week-old, *35S:GFP-ATERF1* plants were infiltrated with OD_600_ = 0.02 of *Pst* strain, harvested after 3–4 h and frozen in liquid nitrogen. Next, 10 g. of plant material were ground to fine powder by using a mortar and pestle and resuspended in 30 ml of NIB buffer (CelLytic™PN, SIGMA), 1 mM DTT. The suspension was filtered and centrifuged at 1260 *g* for 10 min. Pellets were completely resuspended in 1 ml of NIBA (1× NIB, 1 mM DTT and 1% Protease Inhibitor Cocktail), 0.3% TRITON X-100, and centrifuged at 12,000 *g* for 10 min. The supernatants were collected as the cytosolic fractions whereas pellets were washed in 1 ml of NIBA for crude nuclei preparation. Protein concentration for each fraction was determined by the Bradford Protein Assay (Bio-Rad). Sixty µg of protein were loaded per well onto a 10% SDS-PAGE gel and transferred to an Inmobilon™-P PVDF membrane (Millipore). Membranes were blocked in PBS-T containing 5% ECL Advance Blocking Agent (Amersham) and probed with anti-GFP monoclonal (JL-8) antibodies (Living Colors® A.v., Clontech). To monitor for the cleanness of fractions control blots were performed with antibodies against nuclear H3 histone (Abcam) or cytoplasmic RuBisCo-Large subunit (Agrisera). Membranes were incubated with the corresponding secondary antibodies (ECL-peroxidase, Amersham) and chemi-luminescence signals produced with the ECL Advance Western Blotting Detection Kit (Amersham). Signals were detected with the Intelligent Dark-Box II, LAS-1000 scanning system (Fujifilm).

### Production of recombinant ATERF-1 protein

To produce rATERF-1 the full-length CDS of At4g17500 was PCR-up with the PETAT1Fw (5′ CACCATGTCGATGACGGCGGATTC 3′) and PETAT1Rv (5′ TCAAAATTATAAAACCAATAAACGATCGCC3′) primers and the pU16643 DNA as the template, to amplify an 817 bp fragment which was directionally cloned into pET100/D-TOPO expression vector (Champion™ pET Direccional TOPO Expression Kit, Invitrogen). The expression of recombinant protein was induced with 1 mM IPTG in BL21 Star™(DE3) cells of *E. coli* following the procedures recommended by Invitrogen. rLACZ was produced from the pET100/D/lacZ plasmid provided in the kit as the expression control. Purification of rATERF-1 or rLACZ proteins was performed in parallel under native condition, as recommended in Protino NI-IDA Columns user manual (Macherey-Nagel). Recombinant proteins were purified from the soluble fraction obtained after 24 h IPTG-induction and subsequent treatment of the cells with lysozyme (Macherey-Nagel). Both proteins were obtained with a similar purity and maximal concentration (0.2 mg/ml) from the 5^th^ eluted fraction in 1× Protein Elution Buffer (50 mM NaH_2_PO_4_, 300 mM NaCl, 250 mM imidazole pH 8.0).

## Supporting Information

Information S1 **Gene elements spotted on** ***A. thaliana*** **microarrays that were detected in T7LAtPs and T7LATPa libraries.** Graphs represent the number of gene elements that produced signal-to-background ratios below the values specified in the X-axe. The red line shows the threshold ratio produced from hybridization with control elements (genome aliens). A total of 4,274 or 15,390 out of 29,110 elements were below the threshold (non-detected). The numbers above represent gene elements detected in the libraries.
(TIF)

Information S2 **(A) Competition between T7-ATERF-1 and T7-C1 phage for binding to LPS.** Mixtures of phage containing 1∶1 (input 1), 1∶6 (input 2) and 1∶17 (input 3) of T7-ATERF-1:T7-C1 clones were panned against 2.5 mM of agarose-coupled LPS. Uncoupled agarose (Agar) was used as the control for non-specific binding. **(B) Competition between T7-ATUBA1 and T7-C1 phage for binding to** ***Pa*** **,** ***Pst*** **(** ***avrRpt2*** **) (** ***avr*** **) or** ***Pst*** **strains.** Input 1 contains 57.3% of T7-ATUBA1 clone, whereas input 2 contains 48.9%. Asterisks indicate significant differences (*t*-test, p<0.05) respect to the agarose (Agar) control.
(TIF)

Information S3 **Microarray analysis of** ***Pa*** **bio-panning.** Normalized values for log B/L signals.
(TXT)

Information S4 **Microarray analysis of** ***Pst*** **(** ***avrRpt2*** **) bio-panning.** Normalized values for log B/L signals.
(TXT)

Information S5 **Microarray analysis of** ***Pst*** **bio-panning.** Normalized values for log B/L signals.
(TXT)

Information S6 **Significance analysis of microarrays, p-values and fold-changes for 26,450 probes.** Fold-change (FC) is provided as the absolute value, up or down-regulated. Bio-panning rounds 1 to 5 (1B to 5B) against *Pa*, *Pst* or *Pst*(*avrRpt2*) (*avr*) strains.
(XLS)

Information S7 **101 significant genes selected from** ***Pa*** **bio-panning.**
(TXT)

Information S8 **153 significant genes selected from** ***Pst*** **(** ***avrRpt2*** **) bio-panning.**
(TXT)

Information S9 **318 significant genes selected from** ***Pst*** **bio-panning.**
(TXT)

Information S10 **Corrected p-values and fold-change (FC) for the 472-gene set.** p-values before and after FDR correction are shown for each round of selection.
(XLS)

Information S11 **58-gene set shown in** **Figure 7A** **.**
(TXT)

Information S12 **93-gene set shown in** **Figure 7A** **.**
(TXT)

Information S13 **96-gene set shown in** **Figure 7A** **.**
(TXT)

Information S14 **220-gene set shown in** **Figure 7A** **.**
(TXT)

Information S15 **2-gene set shown in** **Figure 7A** **.**
(TXT)

Information S16 **3-gene set shown in Figure A.**
(TXT)

Information S17 **Genes in the 472-gene set that are also transcriptionally regulated upon pathogen challenge.**
(XLSX)

Information S18 **Proteins in the 472-gene set that produced immune interactions in PPIN-1.**
(TXT)
