# Supplementary figures and images for: Wide Screening of Phage-Displayed Libraries Identifies Immune Targets in Planta
Source: PLoS One. 2013 Jan 25;8(1):e54654. doi: 10.1371/journal.pone.0054654 (PMC3556032; doi:10.1371/journal.pone.0054654)

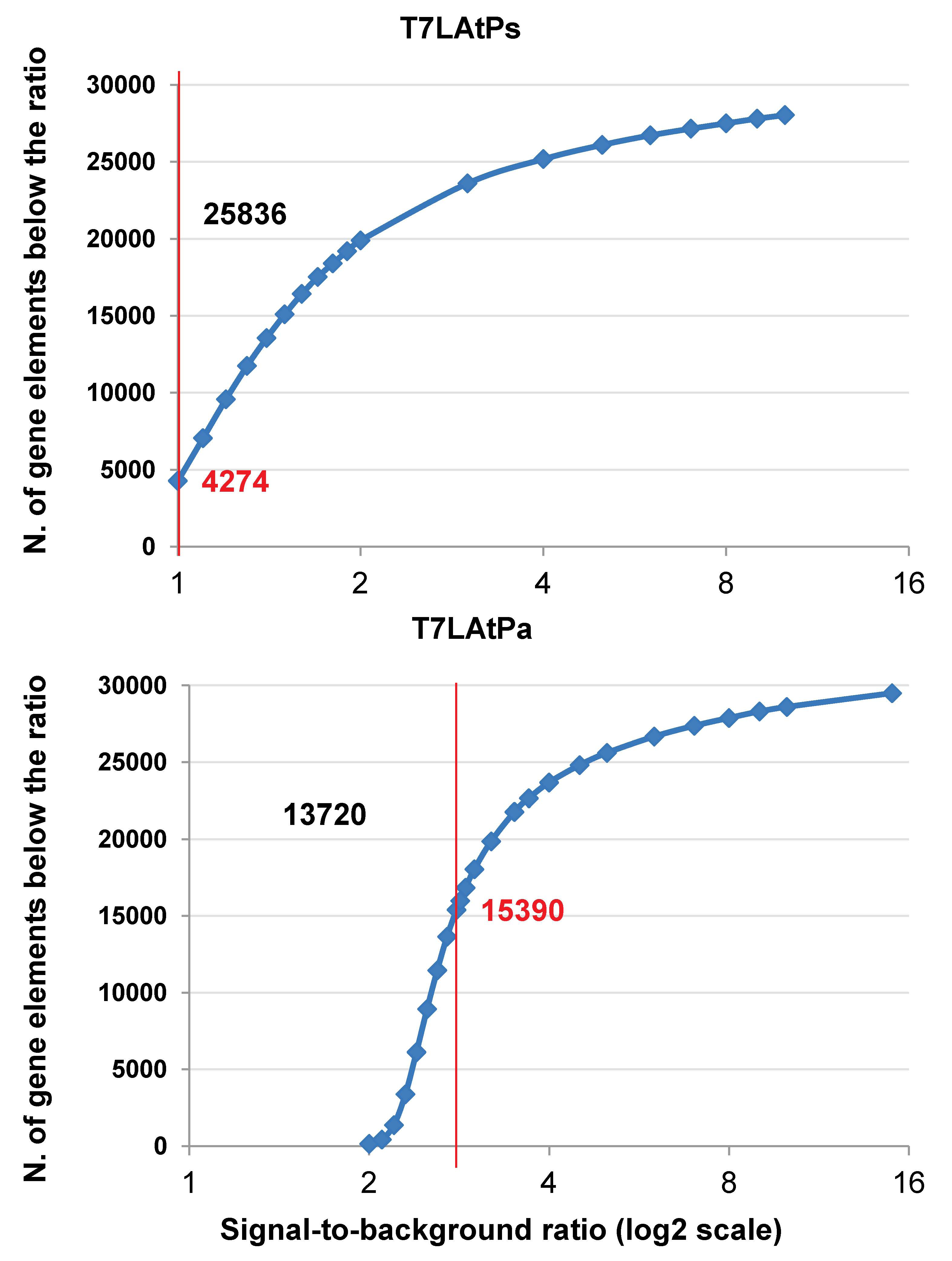

Supplement: Information S1 — Gene elements spotted on A. thaliana microarrays that were detected in T7LAtPs and T7LATPa libraries. Graphs represent the number of gene elements that produced signal-to-background ratios below the values specified in the X-axe. The red line shows the threshold ratio produced from hybridization with control elements (genome aliens). A total of 4,274 or 15,390 out of 29,110 elements were below the threshold (non-detected). The numbers above represent gene elements detected in the libraries. (TIF) [file pone.0054654.s001.tif]

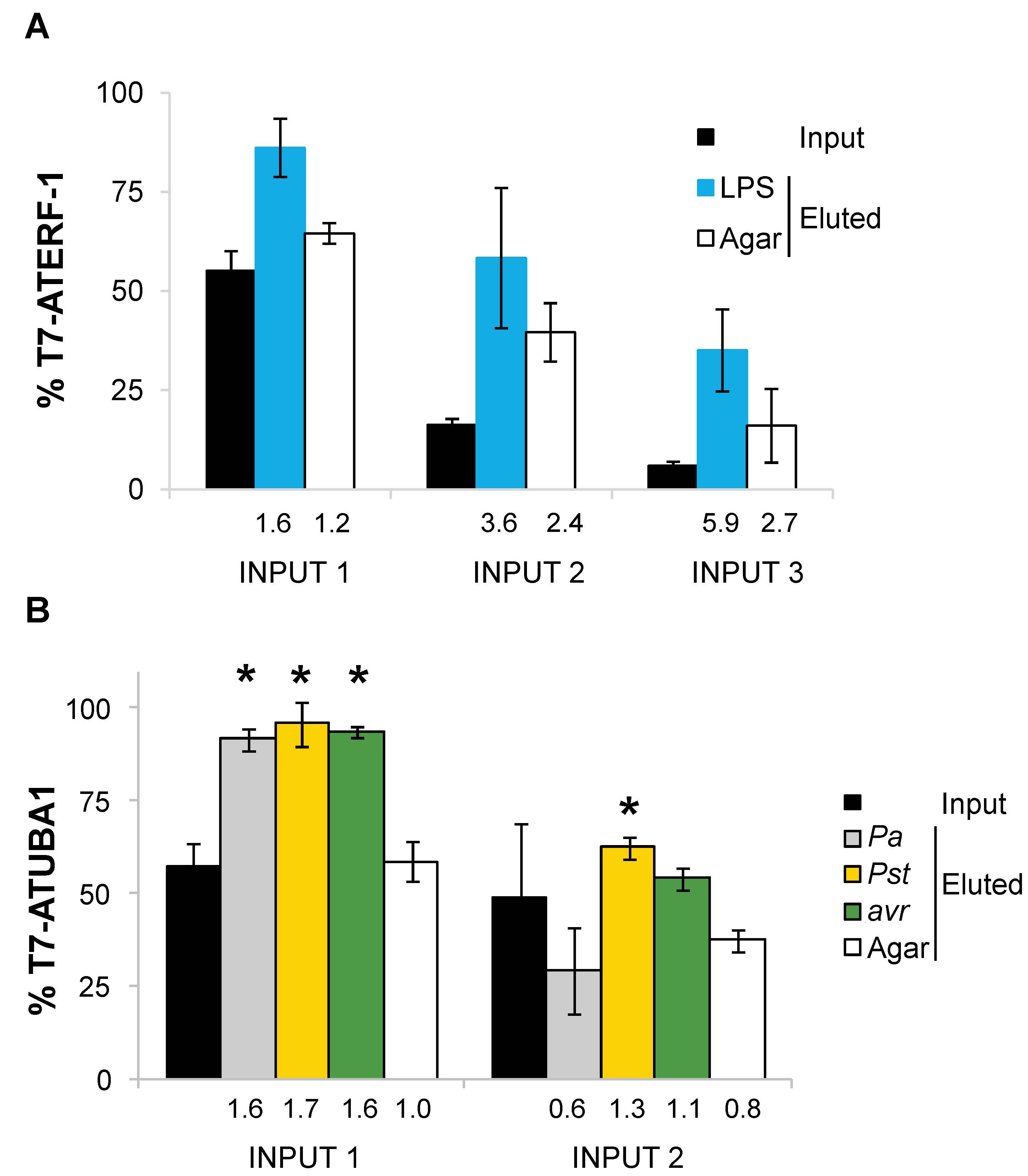

Supplement: Information S2 — (A) Competition between T7-ATERF-1 and T7-C1 phage for binding to LPS. Mixtures of phage containing 1∶1 (input 1), 1∶6 (input 2) and 1∶17 (input 3) of T7-ATERF-1:T7-C1 clones were panned against 2.5 mM of agarose-coupled LPS. Uncoupled agarose (Agar) was used as the control for non-specific binding. (B) Competition between T7-ATUBA1 and T7-C1 phage for binding to Pa , Pst ( avrRpt2 ) ( avr ) or Pst strains. Input 1 contains 57.3% of T7-ATUBA1 clone, whereas input 2 contains 48.9%. Asterisks indicate significant differences (t-test, p<0.05) respect to the agarose (Agar) control. (TIF) [file pone.0054654.s002.tif]
